# Directly visualizing carrier transport and recombination at individual defects within 2D semiconductors†

**DOI:** 10.1039/d0sc07033e

**Published:** 2021-02-09

**Authors:** Joshua W. Hill, Caleb M. Hill

**Affiliations:** Department of Chemistry, University of Wyoming, 1000 E University Ave Laramie WY 82071 USA caleb.hill@uwyo.edu

## Abstract

Two-dimensional semiconductors (2DSCs) are promising materials for a wide range of optoelectronic applications. While the fabrication of 2DSCs with thicknesses down to the monolayer limit has been demonstrated through a variety of routes, a robust understanding of carrier transport within these materials is needed to guide the rational design of improved practical devices. In particular, the influence of different types of structural defects on transport is critical, but difficult to interrogate experimentally. Here, a new approach to visualizing carrier transport within 2DSCs, Carrier Generation-Tip Collection Scanning Electrochemical Cell Microscopy (CG-TC SECCM), is described which is capable of providing information at the single-defect level. In this approach, carriers are locally generated within a material using a focused light source and detected as they drive photoelectrochemical reactions at a spatially-offset electrolyte interface created through contact with a pipet-based probe, allowing carrier transport across well-defined, µm-scale paths within a material to be directly interrogated. The efficacy of this approach is demonstrated through studies of minority carrier transport within mechanically-exfoliated n-type WSe_2_ nanosheets. CG-TC SECCM imaging experiments carried out within pristine basal planes revealed highly anisotropic hole transport, with in-plane and out-of-plane hole diffusion lengths of 2.8 µm and 5.8 nm, respectively. Experiments were also carried out to probe recombination across individual step edge defects within n-WSe_2_ which suggest a significant surface charge (∼5 mC m^−2^) exists at these defects, significantly influencing carrier transport. Together, these studies demonstrate a powerful new approach to visualizing carrier transport and recombination within 2DSCs, down to the single-defect level.

## Abstract 2

Probe-based electrochemical techniques can be used to map carrier transport and recombination within two-dimensional semiconductors.

## Graphical abstract

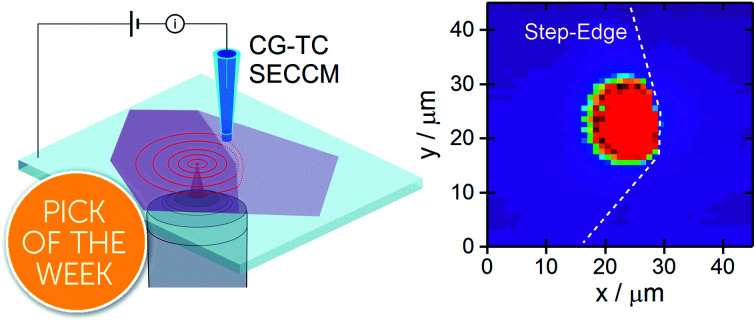

## Introduction

The unique optoelectronic properties of two-dimensional semiconductors (2DSCs), such as the transition metal dichalcogenides (TMDs), make them attractive candidates for use in a variety of electronic and photonic devices, including photovoltaic cells,^1–7^ photodetectors,^8–10^ and LEDs.^11,12^ The inherent 2D structure of these materials allows them to be prepared as ultrathin films down to the monolayer limit, which can serve as flexible active layers with favorable optical properties as compared to the bulk material.^13–18^ Unfortunately, the fabrication of efficient, practical optoelectronic devices based on 2DSCs remains difficult due to an incomplete understanding of the factors governing carrier generation, transport, and recombination in these materials. In particular, the roles played by various types of structural or chemical defects (step edge sites, basal plane vacancies/substitutions, *etc.*) are not yet completely understood. Such defects, whether introduced within a material during synthesis or at interfaces within a device, are known to significantly influence device performance, often serving as detrimental recombination centers.^19–21^

Detailed insights into the behavior of 2DSCs are often difficult to generate due to their heterogeneous structures, which exhibit a variety of defects distributed randomly throughout the material. Traditional characterization techniques produce data which reflects both the bulk properties of a material and collective effects from any defects present within a particular sample. Clearly distinguishing the behavior of defects from that of the bulk material will require high-resolution imaging techniques capable of probing carrier transport and recombination, and a variety of experimental strategies along these lines have been demonstrated. Techniques such as scanning photocurrent microscopy,^22–30^ scanning near-field optical microscopy,^31,32^ electron beam induced current measurements,^33–35^ or transient absorption microscopy^36–40^ have been employed to generate valuable insights into the transport and recombination of carriers within 2DSCs. However, these experiments are often limited in terms of the complexity of the generated response or by the need for carriers to exhibit a strong spectroscopic signature.

Here, we demonstrate that detailed insights into carrier transport and recombination within 2DSCs can be generated using simple, steady-state electrochemical measurements. In this approach, Scanning Electrochemical Cell Microscopy (SECCM) is employed to image the rate of an electrochemical reaction occurring in the vicinity of a localized excitation, directly reflecting the spatial distribution of photogenerated carriers. SECCM utilizes small, electrolyte-filled pipets as probes, creating miniaturized electrochemical cells.^41–55^ By creating and interrogating a series of these cells in a “hopping-mode” fashion, images are constructed which reveal variations in the local electrochemical behavior of a sample. SECCM has been successfully employed to study the catalytic and photoelectrochemical properties of a variety of materials, including 2DSCs.^56–59^ In the studies presented here, we demonstrate a new “Carrier Generation-Tip Collection” (CG-TC) mode of SECCM designed to quantify carrier diffusion lengths within semiconducting materials and locally probe recombination at individual, well-defined defects. This method is applied to visualize carrier transport within mechanically-exfoliated n-WSe_2_ nanosheets, directly revealing the distance photogenerated holes travel within this material and the dramatic impact individual nanoscale defects can have on transport.

## Experimental methods

### Materials and chemicals

I_2_ (Mallinckrodt, U.S.P grade) and NaI (Sigma Aldrich, ≥ 99%) were obtained from the indicated sources and employed without further purification. Ag wire (Alfa-Aesar, 0.25 mm, 99.99%) was utilized as a counter electrode for probe fabrication, and stored in an aqueous solution containing 100 mM NaI and 10 mM I_2_ when not in use. Indium tin oxide (ITO)-coated cover glass slides (22 × 22 mm, #1.5, 30–60 Ω sq.^−1^, SPI) were employed as sample substrates. Bulk n-type WSe_2_ crystals with dopant densities of ∼10^17^ cm^−3^ prepared *via* chemical vapor transport methods^74–76^ were donated by Prof. Bruce Parkinson.

### Sample preparation and characterization

ITO slides were cleaned *via* sequential sonication in isopropanol and deionized (DI) H_2_O. n-WSe_2_ nanoflakes were prepared *via* mechanical exfoliation from bulk crystals and transferred onto ITO substrates using PDMS tape (Gel-Pak, Gel-Film, Pf-40/17-X4). AFM measurements were conducted on a Cypher ES AFM in tapping mode using standard probes (Nanosensors, PPP-NCHR-20, n-Si, 0.01–0.02 Ω cm).

### Probe fabrication and characterization

Pipet-based electrochemical probes were fabricated from quartz capillaries (1.2 mm outer diameter, 0.9 mm inner diameter, Sutter) using a laser-based pipet puller (Sutter P-2000). Probes were fabricated by employing the following two-line program: heat = 750, fil = 4, vel = 30, delay = 135, pull = 80/heat = 685, fil = 3, vel = 30, delay = 135, pull = 150. These probes were filled with an aqueous electrolyte solution containing 0.1 M NaI and 0.01 M I_2_, and a AgI-coated Ag wire was then inserted into the back end of the pipette, completing the probe. The Ag/AgI wire provided a well-defined reference potential in the electrolyte solution corresponding to the AgI + e^−^ → Ag + I^−^ couple. All data provided here is referenced *vs.* this potential. An FEI Quanta FEG 450 field emission scanning electron microscope operating at 5 keV was used for pipette characterization.

### CG-TC SECCM measurements

Samples were mounted onto the stage of an inverted optical microscope. Electrical contacts to the sample were made using Cu tape. The electrochemical probe was mounted to a 3-axis piezoelectric stage (PI P-611.3S). A patch clamp style amplifier was employed to apply an electrical bias between the sample and the Ag/AgI counter electrode and measure the resulting current flow. The sample was placed under focused laser illumination (NA = 0.5, 633 nm, 0.60 µW) and the electrochemical probe was brought to the sample surface while a potential difference of −0.5 V *vs.* Ag/AgI was held at the substrate and the current flowing in the system was monitored. After probe-sample contact was established (indicated by a current spike), the probe was stopped, and a triangular potential waveform (2000 mV s^−1^) was applied, during which the current flowing was recorded. Upon completion of the waveform, the probe was retracted and moved to another location. This process was repeated across a rectangular array of points and the resulting current data was stored as a two-dimensional array with M rows and N columns, where M is the total number of interrogated points and N is the number of current measurements in each voltammogram. All instrumentation was controlled through custom LabView software and a National Instruments DAQ interface (cDAQ-9174). Photocurrent images and voltammograms at specific points of interest were generated from the raw data *via* custom Python scripts.

## Results and discussion

### Methodology

**Fig. 1:**
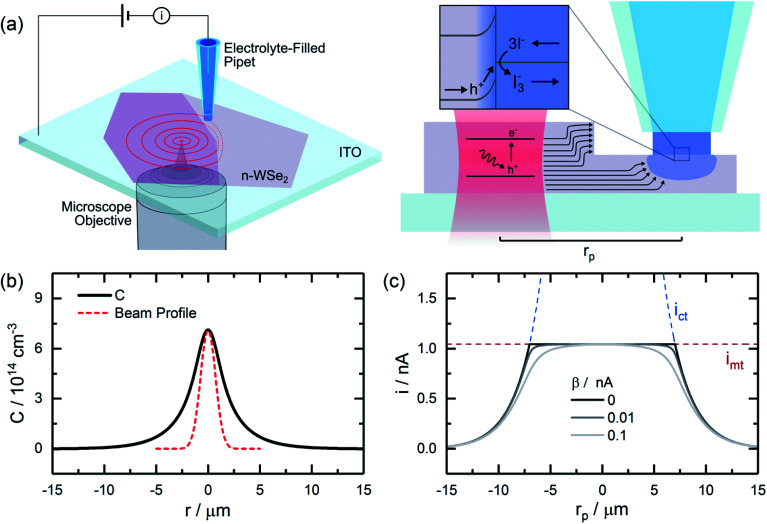
Carrier Generation-Tip Collection Scanning Electrochemical Cell Microscopy (CG-TC SECCM). (a) Minority carriers are generated within a semiconductor through localized illumination and diffuse outward within the material. As carriers reach a probe positioned some distance away, they can be utilized to drive an electrochemical reaction. (b) Idealized 2D carrier profile resulting from illumination with a Gaussian beam. (c) Expected CG-TC SECCM response corresponding to this carrier profile. Dashed lines represent limits corresponding to carrier transport within the semiconductor (*i*_ct_) and the mass transfer of redox species in the electrolyte solution (*i*_mt_). The calculations in (b) and (c) were carried out for a system with *N*_0_ = 3.2 × 10^12^ s^−1^, *t*_c_ = 1 ns, *α* = 10^5^ cm^−1^, *σ*_0_ = 0.725 µm, *w* = 50 nm, *L* = 3 µm, *r*_0_ = 150 nm, *θ*_p_ = 10°, *D*_r_ = 2 × 10^−5^ cm^2^ s^−1^, and 
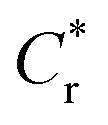
 = 100 mM.

The continuous, localized illumination of a semiconductor will generate steady-state carrier profiles in the vicinity of the excitation. For a Gaussian excitation beam, the concentration profile resulting from diffusive transport in the 2D limit (*i.e.*, for very thin sheets) can be approximated as (see ESI† for details):1
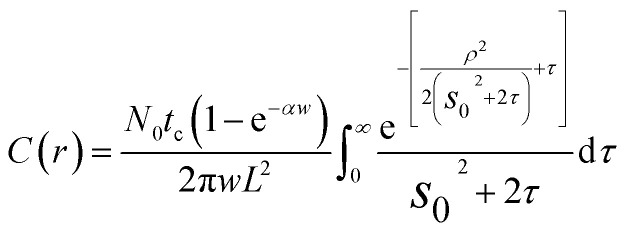
here, *N*_0_ represents the power of the beam (in photons s^−1^), *t*_c_ is the carrier lifetime, *L* is the carrier diffusion length, *α* is the absorption coefficient, *w* is the sheet thickness, *ρ* is a normalized radial distance (*r*/*L*), and *s*_0_ is the normalized standard deviation of the beam profile (*σ*_0_/*L*). Within pristine, defect-free basal planes of a 2D semiconductor, minority carrier profiles should be expected to roughly follow the expression in (1), a plot of which is given in Fig. 1b. These profiles contain valuable information about carrier transport, as their widths will be directly related to the diffusion length of the carriers, 
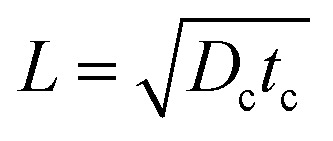
. Near defects which serve as recombination centers, such as steps between adjacent basal planes, these idealized profiles would be altered, introducing anisotropies which reflect the rate at which carriers are transported to the defects and recombine.

Here, SECCM is employed as a tool to locally interrogate minority carrier profiles within 2DSCs, generating valuable information on “bulk” transport within pristine regions of a material and recombination processes at well-defined structural defects. In this approach, referred to here as carrier generation-tip collection (CG-TC) SECCM (Fig. 1a), a small, well-defined electrochemical interface is created by contacting the surface of a semiconductor with an electrolyte-filled pipet. The application of a bias across the semiconducting material and a counter electrode in the electrolyte creates a space charge layer which extends into the material from this interface. A focused laser beam is used to locally generate carriers through photoexcitation of the semiconductor, which then assume spatial profiles similar to those described above. Carriers which reach the boundary of this space charge layer *via* diffusion will be accelerated towards the solid–electrolyte interface and utilized to drive a photoelectrochemical reaction, the rate of which is recorded as the current flowing in the SECCM cell. Provided the probe dimensions are small in comparison to the carrier diffusion length, *L*, this scheme can directly generate information on the spatial distribution of carriers within 2DSCs. Instrumentation schematics and example probe images are provided in the ESI (Fig. S1†).

The resulting CG-TC SECCM currents will depend on the local carrier concentration profile, as well as the kinetics of charge transfer at the solid–electrolyte interface and the mass transfer of redox active species in the electrolyte solution. At steady-state, the rates of each process must be equal, and the overall current can be expressed as (see ESI† for details):2
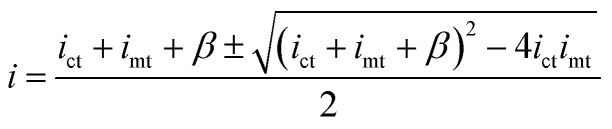
3
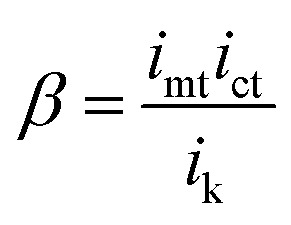
here, *i*_ct_, *i*_mt_, and *i*_k_ represent the currents which would be measured if the system was dictated solely by carrier transport within the semiconductor, mass transport of redox species in the electrolyte, and heterogeneous charge transfer at the interface, respectively. *β* can be viewed as a constant parameter which accounts for the importance of kinetic effects. Assuming purely 2D transport within the semiconductor, these limiting currents can be expressed as:4
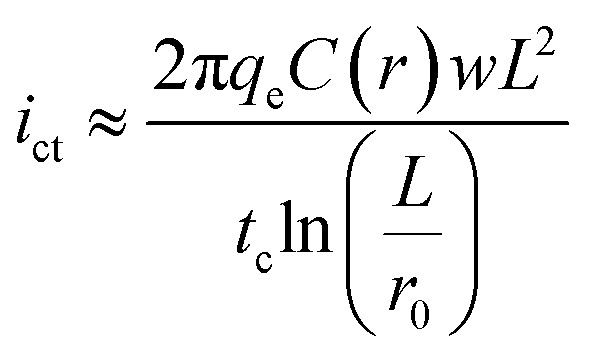
5
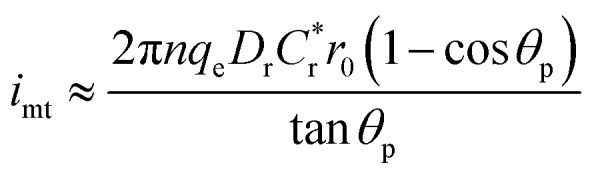
6
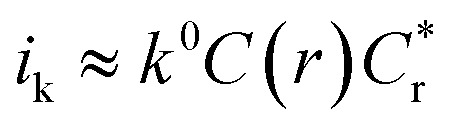
here, q_*e*_ is the elementary charge, *r*_0_ is the probe radius, *θ*_p_ is the half-angle of the probe, *n* is the number of electrons transferred in the photoelectrochemical reaction, *D*_r_ is the diffusion constant for the redox active species, 
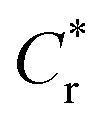
 is the bulk concentration of the redox active species, and *k*^0^ is the heterogeneous rate constant associated with the photoelectrochemical reaction (with units of cm^4^ s^−1^).

An idealized CG-TC SECCM response in the absence of recombination effects (*i.e.*, within an inert basal plane) based on these expressions is depicted in Fig. 1c. Three regions can generally be distinguished based on the excitation-probe distance (*r*_p_). Far away from the excitation centroid, currents will be limited by carrier transport to the electrolyte interface (*i* ≈ *i*_ct_). Near the excitation centroid, currents are limited by the mass transfer of redox species in solution to the interface (*i* ≈ *i*_mt_). The shape of the zone between these limits will be influenced by the heterogeneous kinetics of the reaction, becoming more abrupt with increasing kinetic facility (*β* → 0). As the transition between these regions will be dictated by the relative magnitudes of *i*_ct_ and *i*_mt_ (the latter of which depends on the probe geometry in a well-defined manner), analysis of these “top hat” profiles will allow for direct, quantitative insights into carrier transport. These analytical expressions describe the complete CG-TC SECCM response at a qualitative level, but are only rigorously valid in the 2D limit (*i.e.*, for vanishingly thin sheets). Finite element simulations of carrier transport were utilized to quantitatively analyze CG-TC SECCM data in the studies presented below, and are described in detail in the ESI.†

### Carrier transport within pristine basal planes

**Fig. 2:**
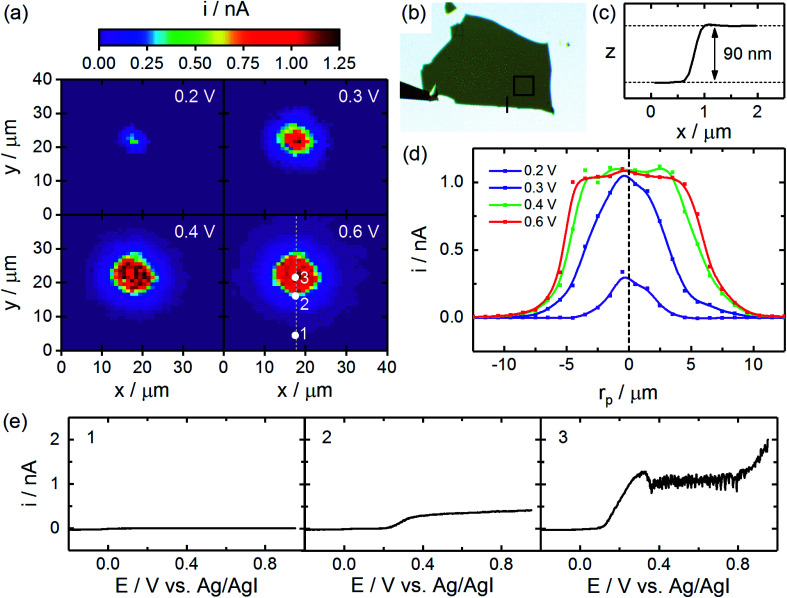
CG-TC SECCM imaging of carrier transport within basal planes of n-WSe_2_ nanosheets. (a) Photocurrent images at a series of different applied potentials (1 µm resolution). (b) Optical transmission image of the n-WSe_2_ nanosheet with the area imaged *via* SECCM and location of the AFM height profile indicated. (c) AFM height profile of the n-WSe_2_ nanosheet. (d) Cross-sections of the SECCM photocurrent images given in (a). (e) Example voltammograms obtained at different points within the imaged area. SECCM data was acquired using a pipet (*d* = 400 nm, *θ*_p_ = 10°) filled with an aqueous solution containing 100 mM NaI, 10 mM I_2_ at a sweep rate of 2000 mV s^−1^. Imaging was carried out in the vicinity of a 633 nm Gaussian beam with *P*_0_ = 600 nW and *σ*_0_ = 0.73 µm.

CG-TC SECCM was first employed to visualize carrier transport within individual basal planes of n-type WSe_2_ nanosheets. Mechanically exfoliated n-WSe_2_ nanosheets were prepared on ITO substrates *via* established techniques and basal planes within these structures were identified *via* optical microscopy. Small (∼300 nm diameter) pipets were then employed to carry out CG-TC SECCM imaging within these basal planes, taking measurements across an array of points spanning a focused excitation source (633 nm laser). Pipets were filled with an aqueous electrolyte containing 100 mM NaI and 10 mM I_2_, allowing photogenerated holes in the n-WSe_2_ nanosheets to drive the oxidation of I^−^ at the electrolyte interface:7
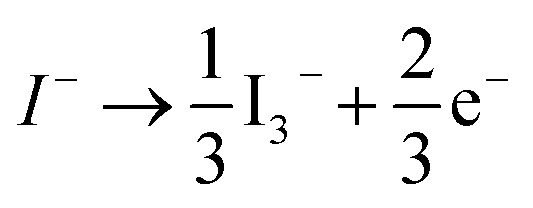
which reflects the electrochemical oxidation of iodide to iodine and the eventual homogeneous formation of triiodide. Results from a representative experiment are shown in Fig. 2. An optical image of the interrogated nanosheet, with a thickness of 90 nm, is given in Fig. 2b. CG-TC SECCM imaging produces a series of photocurrent images which reflect how the rate of the above reaction changes as a function of probe location. A small subset of these images is provided in Fig. 2a; full photocurrent “movies” were also generated from the CG-TC SECCM data, examples of which are provided in the ESI.† In the photocurrent images, symmetric features are observed centered on the excitation centroid. These features grow in size as increasingly anodic potentials are applied, forming a depletion layer which drives photogenerated holes to the electrolyte interface. The size of these features eventually saturates potentials positive of ∼0.6 V *vs.* the reference employed, at which point the response becomes limited by the diffusion of holes to the boundary of the depletion region within the n-WSe_2_ nanosheet, which is confined within ∼100 nm of the electrolyte interface. Alternatively, these data can also be visualized in terms of photocurrent onset potentials through analysis of the point-by-point voltammograms, examples of which are given in Fig. S2 in the ESI.†

Cross-sections of these photocurrent images are provided in Fig. 2d, which clearly show the responses obtained at anodic potentials resemble the idealized, “top hat” response depicted in Fig. 1c. Currents of ∼1 nA are observed in the flat region of the response, which is consistent with the expected mass transfer limit based on diffusion (eqn (5), *n* = 2/3, *D* = 2 × 10^−5^ cm^2^ s^−1^) and suggests migration does not significantly impact the mass transfer of I^−^ in these experiments. Additionally, these data are not significantly affected by *iR* drops, due to the low currents involved (see ESI† for details). At distances of ∼5 µm, currents begin to decrease significantly due to an insufficient density of carriers to drive the oxidation of iodide at the mass transfer limit. The distance at which this transition occurs therefore holds information on the diffusion length of photogenerated carriers. In the analysis below, we take the radial distance where the current falls to half of its mass transfer limited value, *r*_1/2_, as the key metric. Example voltammograms at representative points in the photoelectrochemical image are provided in Fig. 2e. As would be expected, limiting currents increase and photocurrent onset potentials decrease as the probe nears the excitation centroid. In the vicinity of the excitation centroid, non-ideal features in the photocurrent response are also observed at 0.3 V and >0.8 V *vs.* the Ag/AgI reference. The slight drop in current and subsequent noise at 0.3 V is attributable to the formation of an I_2_ film on the n-WSe_2_ surface at high current densities, an effect which has been described in detail previously by a number of researchers.^60–62^ The increase in current beyond 0.8 V at high illumination intensities can be attributed to the onset of photocorrosion of the n-WSe_2_ nanosheet.

**Fig. 3:**
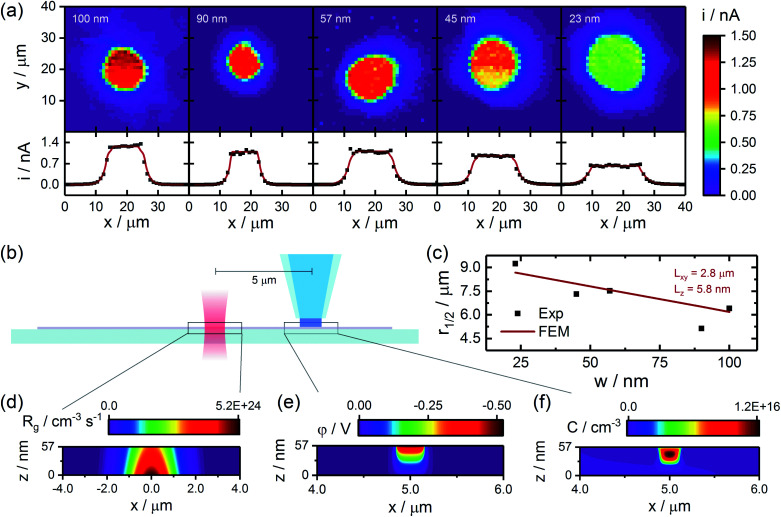
Quantifying carrier transport through analysis of CG-TC SECCM data. (a) CG-TC photocurrent images (0.65 V *vs.* Ag/AgI) and cross-sectional profiles obtained within basal planes of a series of exfoliated n-WSe_2_ nanosheets. Black dots indicate experimental data, and red lines represent finite element simulations. Sheet thickness is indicated in each image. (b) Simplified experimental geometry employed in finite element simulations. (c) Experimental *r*_1/2_ values as a function of sheet thickness and results from finite element simulations for *L*_*xy*_ = 2.8 µm and *L*_*z*_ = 5.8 nm. (d–f) Example steady-state carrier generation, potential, and carrier concentration profiles from finite element simulations. Experimental data was acquired under the same conditions as in Fig. 2. Simulation details are provided in the SI. Note the scales in (d)–(f) are highly anisotropic in order to aid visualization of the results.

While the spatial extent of the observed CG-TC response provides information on carrier transport, 2DSCs like WSe_2_ are known to be highly anisotropic, with transport occurring significantly faster in-plane than out-of-plane. In order to resolve the in-plane and out-of-plane contributions to hole transport in n-WSe_2_, a series of experiments were performed on nanosheets of varying thickness, results from which are summarized in Fig. 3. Photocurrent images obtained within basal planes of a series of nanosheets are given in Fig. 3a, which show the size of the observed CG-TC pattern generally decreases with increasing sheet thickness. This can be attributed to the back-illumination configuration employed in these experiments, making it more difficult for holes to reach the electrochemical interface at larger sheet thicknesses due to slow out-of-plane transport. This effect can be quantitatively expressed in terms of *r*_1/2_ as depicted in Fig. 3c. Finite element simulations were employed to analyze these values, which are described in detail in the ESI.† In these simulations, steady-state solutions to Poisson's equation (describing the potential distribution in the nanosheet) and the drift-diffusion equation (describing carrier transport) were found simultaneously. Carrier transport limited currents (*i*_ct_ values) were calculated from these simulations based on the flux at the pipet interface, and overall currents were calculated from these values following eqn (2). *i*_mt_ values were determined experimentally, and *β* was employed as a variable parameter (though its value did not significantly impact the determination of diffusion lengths).

Example carrier generation (*R*_g_), potential (*φ*), and carrier concentration (*C*) profiles are provided in Fig. 3d–f. Based on these simulations, fields within the nanosheet are confined to within ∼100 nm of the pipet interface. Holes which reach the boundary of this space charge region *via* diffusion are accelerated towards the pipet interface by these fields, contributing to the rate of the photoelectrochemical process. As the size of the space charge region is much smaller than the distance traveled by carriers in these CG-TC experiments (∼5 µm), it can be safely assumed that results from these experiments are dictated largely by diffusion. In-plane (*L*_*xy*_) and out-of-plane (*L*_*z*_) diffusion lengths were thus varied to match the experimental *r*_1/2_ values presented in Fig. 3c, and good agreement between experimental results and simulations was obtained for *L*_*xy*_ = 2.8 µm and *L*_*z*_ = 5.8 nm. Hole transport is thus highly anisotropic, exhibiting a diffusion length ratio of *L*_*xy*_/*L*_*z*_ ≈ 500 (which would correspond to a mobility ratio of ∼2.5 × 10^5^). This ratio of in-plane to out-of-plane minority carrier diffusion lengths is significantly larger than reported values for n-WSe_2_ generated through traditional, bulk photoelectrochemical experiments,^63^ which may be attributable to the degree to which the experimental geometry is defined and the influence of defects can be controlled in the CG-TC SECCM experiments presented here. This value is, however, consistent with the broader range of studies of carrier transport in TMD materials, where mobility ratios up to ∼10^7^ have been reported.^63–68^

### Carrier recombination at individual, nanoscale defects

While CG-TC SECCM experiments carried out within pristine basal planes allow carrier diffusion lengths within single, well-defined nanostructures to be quantified, a potentially more powerful application of this technique lies in interrogating carrier transport across different types of structural defects. Because the excitation source generating carriers and the SECCM probe serving as a collection point can be arbitrarily configured within a structure, transport across individual nanoscale defects can be straightforwardly probed in the CG-TC geometry, allowing recombination effects to be clearly visualized and local recombination rates or transport mechanisms to be quantitatively interrogated.

**Fig. 4:**
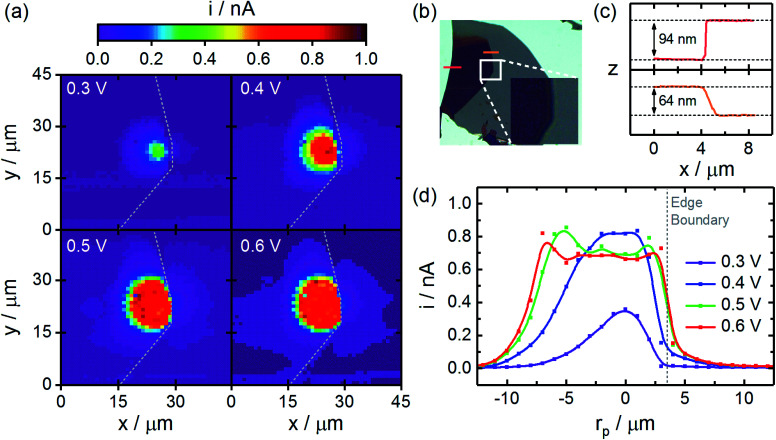
CG-TC SECCM imaging of carrier transport across an individual step edge defect within an n-WSe_2_ nanosheet. (a) Photocurrent images at a series of different applied potentials (1 µm resolution). (b) Optical transmission image of the n-WSe_2_ nanosheet with the area imaged *via* SECCM and locations of the AFM height profiles indicated. (c) AFM height profiles of the n-WSe_2_ nanosheet. (d) Cross-sections of the SECCM photocurrent images given in (a). SECCM data was acquired using a pipet (*d* = 250 nm, *θ*_p_ = 8.5°) filled with an aqueous solution containing 100 mM NaI, 10 mM I_2_ at a sweep rate of 2000 mV s^−1^. Imaging was carried out in the vicinity of a 633 nm Gaussian beam with *P*_0_ = 600 nW and *σ*_0_ = 0.73 µm.

Experiments demonstrating this approach in the n-WSe_2_ system are depicted in Fig. 4, and additional examples are provided in Fig. S3 in the ESI.† Here, CG-TC SECCM imaging was carried out within an n-WSe_2_ nanosheet with the excitation located ∼3 µm from a ∼60 nm step edge. An optical micrograph of the nanosheet is provided in Fig. 4b. Photoelectrochemical reaction rates were mapped across both sides of the step, allowing the density of photogenerated carriers to be probed both near the illuminated region and across the defect. Photocurrent images at various potentials are provided in Fig. 4a. Similar to measurements taken within basal planes, photocurrents increased in magnitude and widened spatially with increasing potential, eventually saturating as the signals become limited by diffusion to the boundary of the space charge region. Within the illuminated side of the step edge, photogenerated holes diffuse isotropically away from the excitation centroid in a similar fashion to the basal plane studies in Fig. 2. However, photocurrents at and across the edge (indicated by the dashed line) were significantly reduced, providing a clear, unambiguous visualization of carrier recombination at a single nanoscale defect. Cross-sectional photocurrent profiles are provided in Fig. 4d, with the relative location of the step edge indicated. Distinctly different shapes are observed here as compared to the basal plane measurements. A significant asymmetry exists within these profiles, with photocurrents increasing more drastically away from the step edge. Photocurrents measured at and across the step edge were significantly lower, reflecting efficient charge carrier recombination at the defect surface. These results do not reflect local variations in the kinetics of I^−^ oxidation, as all measurements are obtained at basal plane surfaces.

**Fig. 5:**
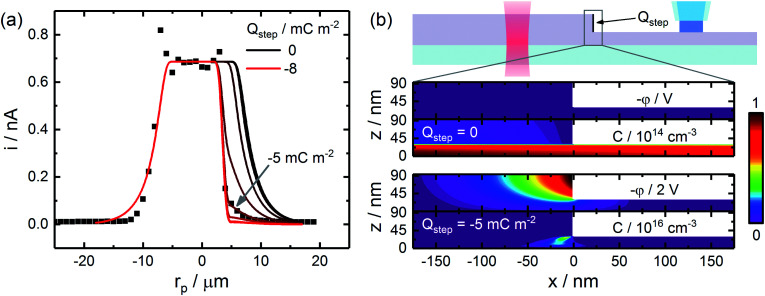
Finite element modeling of hole transport across an individual step edge. (a) Experimental and simulated CG-TC SECCM responses, utilizing diffusion lengths determined from basal plane measurements as inputs. An effective surface charge at the step edge surface was varied between 0 and −8 mC m^−2^ in 1 mC m^−2^ increments. Simulations were carried out for the sheet geometry depicted in Fig. 4, assuming diffusion lengths of *L*_*xy*_ = 2.8 µm and *L*_*z*_ = 5.8 nm. (b) Simulated potential (*φ*) and hole concentration (*C*) profiles in the vicinity of the step edge defect in the absence or presence of a −5 mC m^−2^ surface charge.

Finite element simulations were employed to quantitatively examine recombination at these defects, finding solutions to the drift-diffusion equations while treating the defect surface as an efficient recombination center (hole concentration set to zero). Results from these simulations, which employed the diffusion lengths determined in the basal plane studies, are provided in Fig. 5. Due to the large anisotropy in diffusion lengths, simulations which considered transport to the step edge surface *via* diffusion did not predict step edge defects would exhibit a considerable impact on CG-TC experiments. While holes generated within the “top” section of the structure could be efficiently transported to the defect surface and recombine, carriers produced within the “bottom” section would be largely unaffected due to slow out-of-plane diffusion. In order to explain the drastic limitations in transport observed experimentally, a significant field-driven mechanism must also be considered. This can be accomplished through the inclusion of an effective negative surface charge across the step edge, creating an electric field within the nanosheet which drives transport towards the defect. Results from these simulations suggest that an effective surface charge of *ca.* −5 mC m^−2^ exists across the step edge surface, likely originating from surface oxides which form selectively at these defect-rich sites.^69–73^ Surface oxide layers on WSe_2_ have been shown to exhibit electron trap densities upwards of 10^12^ cm^−2^,^70^ which is consistent with the mC m^−2^-scale surface charges observed here. These results suggest that chemically modifying step edge defects with species which prevent oxide formation or counteract the resulting surface charge may serve as an effective means of mitigating carrier recombination in these materials.

### Carrier confinement within more complex defect geometries

**Fig. 6:**
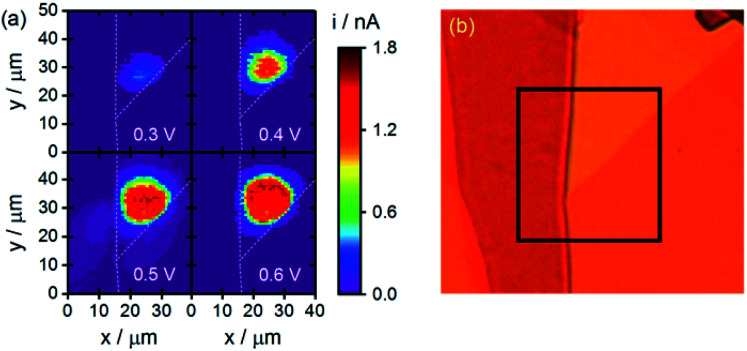
Carrier confinement within more complex defect geometries. (a) CG-TC SECCM photocurrent images with the photoexcitation located near a triangular boundary within an n-WSe_2_ nanosheet. (b) An optical transmission image of the same nanosheet, with the area interrogated *via* SECCM indicated. Experimental parameters were identical to those employed in Fig. 2.

The dramatic reduction in carrier transport across step edge defects observed above would be expected to confine carriers within more complex geometries. An example applying CG-TC SECCM to visualize this confinement is provided in Fig. 6. Here, imaging was carried out within an n-WSe_2_ nanosheet with step edge defects enclosing a triangular area. As before, the obtained SECCM patterns increased with increasing potential, moving radially outward from the excitation centroid. At large potentials, the signals are abruptly halted at each step edge, indicating strong hole confinement due to the presence of these defects. These experiments provide direct, visual confirmation of the inability of carriers to travel over large distances laterally within n-WSe_2_ in the presence of surface defects, further demonstrating the need to develop passivation techniques to mitigate these effects in applications where significant carrier diffusion lengths are necessary.

## Conclusions

In this report, carrier transport within exfoliated n-WSe_2_ nanosheets was investigated using a Carrier Generation-Tip Collection (CG-TC) mode of Scanning ElectroChemical Cell Microscopy. This approach, wherein carriers are generated locally within a focused excitation source and utilized to drive a photoelectrochemical reaction at a spatially-offset probe, enables carrier transport across arbitrarily defined pathways within individual nanostructures to be quantitatively investigated. Analysis of CG-TC SECCM experiments carried out within pristine basal planes of n-WSe_2_ nanosheets of varying thickness revealed in-plane and out-of-plane diffusion lengths of *L*_*xy*_ = 2.8 µm and *L*_*z*_ = 5.8 nm. Experiments investigating carrier transport across well-defined step edge defects provided direct, visual evidence of the dramatic limitations to carrier transport imposed by these features, suggesting a significant surface charge exists which drives the transport of holes to these recombination centers. Together, these experiments demonstrate CG-TC SECCM to be a uniquely powerful tool for investigating carrier transport within 2D semiconducting materials.

## Author contributions

J. W. H. and C. M. H. designed the project. J. W. H. acquired all data. J. W. H. and C. M. H. analyzed data, performed simulations, and wrote the paper.

## Conflicts of interest

There are no conflicts to declare.

## Supplementary Material

SC-012-D0SC07033E-s001

SC-012-D0SC07033E-s002

SC-012-D0SC07033E-s003

SC-012-D0SC07033E-s004
