# Supplementary material for: Directly visualizing carrier transport and recombination at individual defects within 2D semiconductors
Source: Chem Sci. 2021 Feb 9;12(14):5102–12. doi: 10.1039/d0sc07033e (PMC8179556; doi:10.1039/d0sc07033e)
Supplement: SC-012-D0SC07033E-s004 [file SC-012-D0SC07033E-s004.pdf]

Electronic Supplementary Material (ESI) for Chemical Science.
This journal is © The Royal Society of Chemistry 2021

Supporting Information

Directly Visualizing Carrier Transport and Recombination at Individual Defects within 2D Semiconductors

Joshua W. Hill and Caleb M. Hill*
Department of Chemistry, University of Wyoming, Laramie, WY 82071
*caleb.hill@uwyo.edu

Table of Contents

Theoretical Derivations 1
    Definition of Mathematical Symbols 1
    Steady-State Minority Carrier Profile 2
    Carrier-Transport Limited SECCM Currents in 2D 4
    Mass Transfer-Limited Currents in the SECCM Geometry 5
    Steady-State Model for CG-TC SECCM Responses 5
    Estimation of iR Drops in the SECCM Geometry 6
Additional Experimental Data 7
Details on Finite Element Simulations 8
    Theoretical Framework 8
    Values of Physical Constants Employed in Finite Element Simulations 9
References 9
COMSOL Model Report 10

Theoretical Derivations

Definition of Mathematical Symbols

Symbol	Definition
ϵ	Dielectric Constant
χ	Dimensionless Carrier Concentration, $C/R_g t_c$
φ	Electric Potential
τ	Dimensionless Time, $(t - t_0)/t_c$
σ	Solution Conductivity
ρ	Dimensional Radial Distance from Beam Centroid, r/L
ν	Frequency
β	Kinetic Parameter
α	Absorption Coefficient or Constant in Modified Bessel's Equation
w	Layer Thickness
t	Time
r	Radial Distance from Beam Centroid
n	Effective # of Electrons in Reaction
m	Mass Transfer Coefficient
j	Current Density
T	Temperature
R	Solution Resistance

Symbol	Definition
Q	Surface Charge Density
L	Carrier Diffusion Length
I	Beam Intensity
E_{fb}	Flat-Band Potential
E	Potential
D	Diffusion Constant
C	Carrier Concentration
h	Planck's Constant
ϵ_0	Vacuum Permittivity
σ_0	Beam Standard Deviation
θ_p	Pipet Half-Angle
y_g	y-centroid of Beam
x_g	x-centroid of Beam
t_c	Carrier Lifetime
t_0	Carrier Generation Time
s_0	Dimensionless Beam Standard Deviation, σ_0/L
r_{sc}	Edge of Space Charge Layer in Semiconductor
r_s	Inner Radius of Spherical Sector Approximating Pipet Geometry
r_0	Pipet Radius
q_e	Electronic Charge
n_D	Dopant Density
k_b	Boltzmann's Constant
k^0	Heterogeneous Rate Constant
i_{mt}	Mass Transport-Limited Current
i_k	Kinetically-Limited Current
i_{ct}	Carrier Transport-Limited Current
R_g	Carrier Generation Rate
P_0	Beam Power
N_0	Beam Power in Photons/s
K_0	Modified Bessel's Function of the Second Kind

Steady-State Minority Carrier Profile

Consider a semiconductor illuminated with a gaussian beam with an intensity which varies spatially as:

$$I = \frac{P_0}{2\pi\sigma_0^2 h\nu} e^{-\frac{(x-x_g)^2+(y-y_g)^2}{2\sigma_0^2}} \qquad S1$$

Where P_0 is the power of the beam, σ_0 is the standard deviation of the gaussian profile, $h\nu$ is the photon energy, and x_g/y_g denote the centroid coordinates. Here, I is given in units of $cm^{-2}\ s^{-1}$. To a first approximation, the generation of carriers within a thin semiconducting layer of thickness w can be expressed as:

$$R_g = \frac{P_0(1 - e^{-\alpha w})}{2\pi\sigma_0^2 h\nu w} e^{-\frac{(x-x_g)^2+(y-y_g)^2}{2\sigma_0^2}} \qquad S2$$

Where α is the absorption coefficient of the semiconductor. Illumination with a short pulse of duration dt_0 results in an initial carrier distribution:

$$dC_0 = \frac{P_0(1 - e^{-\alpha w})dt_0}{2\pi\sigma_0^2 h\nu w} e^{-\frac{(x-x_g)^2+(y-y_g)^2}{2\sigma_0^2}} \qquad S3$$

Within the semiconductor, this distribution will evolve over time according via diffusion, following:

$$\frac{\partial C}{\partial t} = D\nabla^2 C - \frac{C}{t_c} \qquad S4$$

Here, D is the diffusion coefficient of the carriers and t_c is the carrier lifetime. Using S3 as an initial condition results in the following well-known solution:[1]

$$dC = \frac{P_0(1 - e^{-\alpha w})dt_0}{2\pi[\sigma_0^2 + 2D(t - t_0)]h\nu w} e^{-\frac{(x-x_g)^2+(y-y_g)^2}{2[\sigma_0^2+2D(t-t_0)]}} e^{-\frac{(t-t_0)}{t_c}} \qquad S5$$

Here, t_0 denotes the time at which the pulse is generated. An analogous solution for the case of steady-state illumination can be found through the principle of superposition. I.e., by integrating the above expression over all possible pulse times, t_0:

$$C = \frac{P_0(1 - e^{-\alpha w})}{2\pi h\nu w} \int_{-\infty}^{t} \frac{e^{-\frac{(x-x_g)^2+(y-y_g)^2}{2[\sigma_0^2+2D(t-t_0)]}} e^{-\frac{(t-t_0)}{t_c}}}{[\sigma_0^2 + 2D(t - t_0)]} dt_0 \qquad S6$$

Making the substitution $\tau = (t - t_0)/t_c$ and defining a diffusion length as $L = \sqrt{Dt_c}$ yields:

$$C = \frac{P_0(1 - e^{-\alpha w})}{2\pi h\nu w} \int_{0}^{\infty} \frac{e^{-\frac{(x-x_g)^2+(y-y_g)^2}{2[\sigma_0^2+2L^2\tau]}} e^{-\tau}}{[\sigma_0^2 + 2L^2\tau]} d\tau \qquad S7$$

Defining $r^2 = (x - x_g)^2 + (y - y_g)^2$, $N_0 = P_0/h\nu$, and introducing dimensionless parameters $\rho = r/L$, $s_0 = \sigma_0/L$ allows the profile to be written as:

$$C(r) = \frac{N_0 t_c(1 - e^{-\alpha w})}{2\pi w L^2} \int_{0}^{\infty} \frac{e^{-\left[\frac{\rho^2}{2(s_0^2+2\tau)}+\tau\right]}}{s_0^2 + 2\tau} d\tau \qquad S8$$

This expression describes the photogenerated carrier profile in the vicinity of a gaussian excitation source, assuming carriers move solely via 2D diffusion.

Carrier-Transport Limited SECCM Currents in 2D

Consider a thin semiconducting layer which is evenly illuminated (constant R_g). In the absence of applied fields, the generation and transport of carriers within this system can be described via:

$$\frac{\partial C}{\partial t} = D\nabla^2 C - \frac{C}{t_c} + R_g \qquad S9$$

In 2D, steady-state solutions to this equation will satisfy:

$$D\left[\frac{\partial^2}{\partial r^2} + \frac{1}{r}\frac{\partial}{\partial r}\right]C - \frac{C}{t_c} + R_g = 0 \qquad S10$$

Making the substitutions $L = \sqrt{Dt_c}$, $\rho = r/L$, and $\chi = C/R_g t_c$ allows this to be written as:

$$\rho^2\frac{\partial^2\chi}{\partial\rho^2} + \rho\frac{\partial\chi}{\partial\rho} - \rho^2\chi = -\rho^2 \qquad S11$$

This equation is an inhomogeneous variant of the modified Bessel's equation:

$$\rho^2\frac{\partial^2\chi}{\partial\rho^2} + \rho\frac{\partial\chi}{\partial\rho} - (\rho^2 + \alpha^2)\chi = 0 \qquad S12$$

The general solution to the equation will thus be:

$$\chi = 1 + \gamma I_0(\rho) + \delta K_0(\rho) \qquad S13$$

Where I_0 and K_0 are modified Bessel functions of the first and second kind, respectively and γ/δ are constants. Requiring $\chi \to 1$ as $\rho \to \infty$ yields $\gamma = 0$. δ can be evaluated by requiring the concentration to be 0 at ρ_{sc}, which would correspond to the edge of the space charge layer within the semiconductor. The solution is then:

$$\chi = 1 - \frac{K_0(\rho)}{K_0(\rho_{sc})} \qquad S14$$

In explicit terms, the concentration profile under diffusion-limited transport is then:

$$C = R_g t_c\left[1 - \frac{K_0\left(\frac{r}{L}\right)}{K_0\left(\frac{r_{sc}}{L}\right)}\right] \qquad S15$$

The current flowing can be calculated as:

$$i_{ct} = 2\pi q_e D r_0 w\left.\frac{\partial C}{\partial r}\right|_{r=r_{sc}} \qquad S16$$

For small x, $K_0(x) \approx -\ln(x)$, making the flux:

$$\frac{\partial C}{\partial r} \approx \frac{R_g t_c}{r\ln\left(\frac{L}{r_{sc}}\right)} \qquad S17$$

And the current is then:

$$i_{ct} \approx \frac{2\pi q_e R_g w L^2}{\ln\left(\frac{L}{r_{sc}}\right)} \qquad S18$$

Mass Transfer-Limited Currents in the SECCM Geometry

Consider a conical pipet with terminal radius r_0 and half-angle θ_p. The mass transfer-limited currents in this system can be approximated by considering diffusion within a spherical sector which approximates the pipet geometry. The radius of the sector (r_s) subtended by the pipet geometry can be approximated as:

$$r_s = \frac{r_0}{\tan\theta_p} \qquad S19$$

This approximation effectively ignores transport through the meniscus created through pipet contact. Because the system can be approximated in a spherical geometry, the mass transfer coefficient is simply:

$$m = \frac{D_r}{r_s} \qquad S20$$

where D_r is the diffusion coefficient for redox species in solution. The effective surface area is:

$$A = 2\pi\left[1 - \cos\theta_p\right]r_s^2 \qquad S21$$

The current can then be evaluated as:

$$i = nq_e AmC_r^* = \frac{2\pi nq_e D_r C_r^* r_0\left[1 - \cos\theta_p\right]}{\tan\theta_p} \qquad S22$$

where C_r^* is the bulk concentration of redox species in solution.

Steady-State Model for CG-TC SECCM Responses

At steady-state, the rate carrier transport to the space charge layer within the semiconductor, heterogeneous charge transfer at the electrochemical interface, and mass transport of redox species in solution must be equal:

$$i = i_{ct} = i_k = i_{mt} \qquad S23$$

Here, *ct*, *k*, and *mt* refer to charge transport, kinetic, and mass transfer-limited currents. These will be assumed to have the expressions:

$$i = i_{ct} = q_e Am_{ct}[C^* - C] = i_{CT}\left(1 - \frac{C}{C^*}\right) \qquad S24$$

$$i = i_k = q_e Ak^0 CC_r = i_K\left(\frac{CC_r}{C^* C_r^*}\right) \qquad S25$$

$$i = i_{mt} = q_e Am_r[C_r^* - C_r] = i_{MT}\left(1 - \frac{C_r}{C_r^*}\right) \qquad S26$$

Here it is assumed that the carrier transport and mass transfer rates can be described by traditional expressions, being proportional to the difference between bulk and interfacial concentrations. For carrier transport, it is being assumed that the "bulk" concentration changes with pipet position. Combining these expressions and simplifying yields:

$$i^2 - (i_{CT} + i_{MT} + \beta)i + i_{CT}i_{MT} = 0 \qquad S27$$

where a kinetic parameter, β has been defined as:

$$\beta = \frac{i_{mt}i_{ct}}{i_k} \qquad S28$$

This equation has the solutions:

$$i = \frac{(i_{CT} + i_{MT} + \beta) \pm \sqrt{(i_{CT} + i_{MT} + \beta)^2 - 4i_{CT}i_{MT}}}{2} \qquad S29$$

Note that if $\beta \to 0$ (very fast kinetics):

$$i \approx \frac{i_{CT} + i_{MT} \pm \sqrt{(i_{CT} - i_{MT})^2}}{2} = \frac{i_{CT} + i_{MT} \pm (i_{CT} - i_{MT})}{2} = i_{CT} \; or \; i_{MT} \qquad S30$$

Estimation of iR Drops in the SECCM Geometry

Consider again a conical pipet with terminal radius r_0 and half-angle θ_p. The potential profile within this pipet can be approximated as a solution to Poisson's equation for a spherical sector which approximates the pipet geometry:

$$\nabla^2\varphi = 0 \qquad S31$$

where it has been assumed that the charge density everywhere within the pipet volume is zero. Taking the potential of the terminal surface to be zero, and that at large distances to be φ_∞, this has the following solution:

$$\varphi = \varphi_\infty \left[1 - \frac{r_s}{r}\right] \qquad S32$$

where r_s is the radius of the sector as defined before. The current density at any point within the pipet can be found as:

$$j = -\sigma\frac{\partial\varphi}{\partial r} = -\frac{\sigma\varphi_\infty r_s}{r^2} \qquad S33$$

The total current flowing in the pipet can be found by integrating this expression across a spherical surface within the pipet. This is most easily accomplished by multiplying by the area $A = 2\pi\left[1 - \cos\theta_p\right]r^2$:

$$i = jA = -2\pi\left[1 - \cos\theta_p\right]r_s\sigma\varphi_\infty \qquad S34$$

Defining the pipet resistance as $R = -\varphi_\infty/i$ and substituting for r_s, its value is found to be:

$$R = \frac{\tan\theta_p}{2\pi\left[1 - \cos\theta_p\right]r_0\sigma} \qquad S35$$

Taking the conductivity[2] of a 100 mM NaI solution to be 10.9 mS cm^{-1} and assuming a typical pipet geometry of r_0 = 150 nm and θ_p = 10°, $R \approx 10^7$ Ω. Thus, for the nA-level currents observed in the experiments here, iR drops are anticipated to be on the level of ~10 mV and largely inconsequential.

Additional Experimental Data

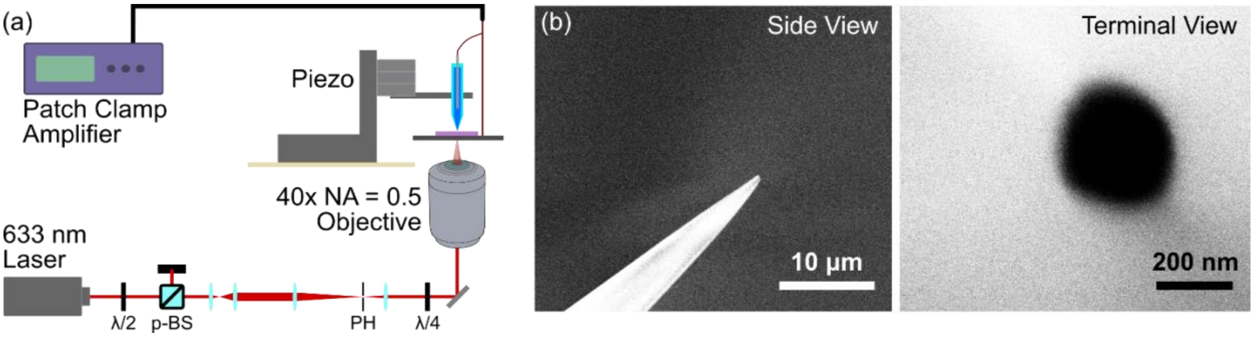

Figure S1. (a) Experimental configuration employed in CG-TC SECCM studies and (b) representative SEM images of a pipet probe employed for SECCM imaging. In (a), $\lambda/2$ = half-wave plate, $\lambda/4$ = quarter-wave plate, p-BS = polarizing beam-splitter, and PH = pinhole. Together, the half-wave plate and beamsplitter act as a continuously variable attenuator.

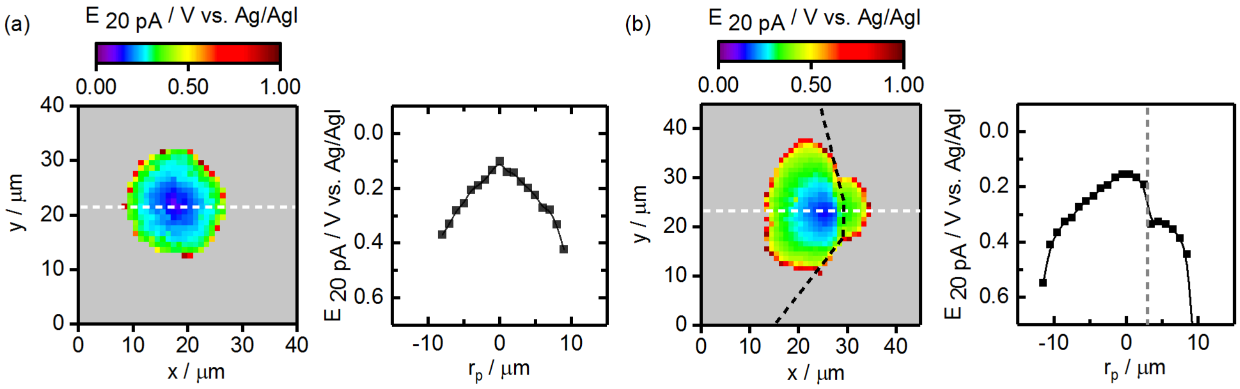

Figure S2. CG-TC SECCM photovoltage images acquired within an n-WSe_2 basal plane (a) and across an individual step-edge defect within an n-WSe_2 nanosheet. The images were constructed by calculating the potential necessary to achieve a photocurrent of 20 pA from the CG-TC SECCM data depicted in **Figures 2** and **4** in the main text.

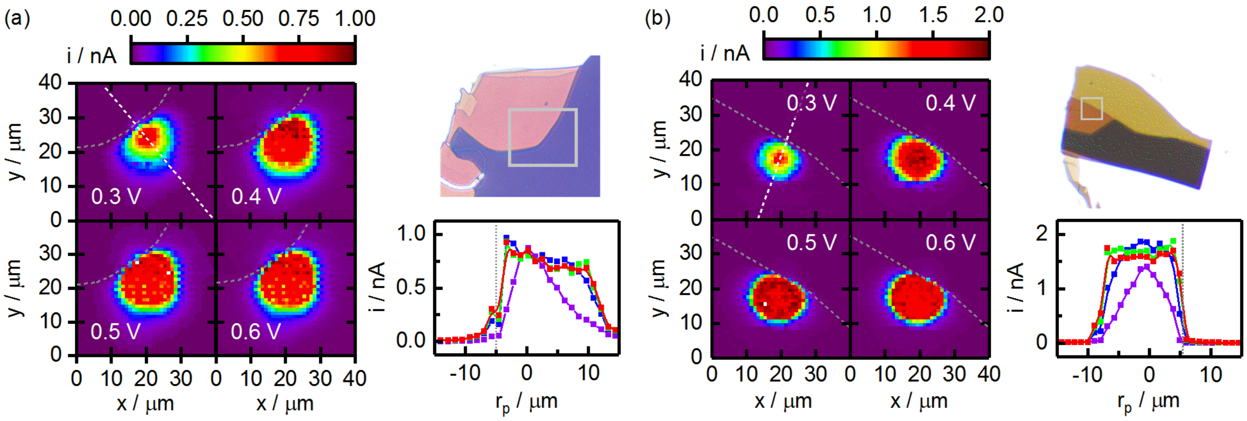

Figure S3. Additional examples of CG-TC SECCM experiments probing carrier transport across individual step-edge defects in n-WSe_2 nanosheets. All data were acquired using an aqueous solution containing 100 mM NaI and 10 mM I_2. Potentials were swept from -1 V to 0.5 V at 2000 mV/s vs. Ag/AgI. Imaging was carried out in the vicinity of a 633 nm Gaussian beam with P_0 = 600 nW and σ_0 = 0.73 μm.

Details on Finite Element Simulations

Theoretical Framework

The approximate treatment outlined above, which considers only 2D transport via diffusion within an infinite semiconducting layer, is only intended to provide qualitative insights into the CG-TC SECCM method. For quantitative insights, finite element simulations were carried out using COMSOL Multiphysics. These simulations were employed to find steady-state solutions to Poisson's equation (which dictates fields within the material) and the drift-diffusion equation (governing carrier transport):

$$\nabla \cdot (\boldsymbol{\epsilon} \nabla \varphi) = \frac{q_e n_D}{\epsilon_0}\left[1 - e^{-\frac{q_e \varphi}{k_b T}}\right] \qquad S36$$

$$\nabla \cdot \left(\boldsymbol{D} \nabla C + \frac{q \boldsymbol{D} \nabla \varphi}{k_b T} C\right) - \frac{C}{t_c} + \frac{\alpha P_0}{2\pi \sigma_0^2 h\nu} e^{-\frac{(x-x_g)^2 + (y-y_g)^2}{2\sigma_0^2}} e^{-\alpha z} = 0 \qquad S37$$

In these equations, $\boldsymbol{\epsilon}$ is the dielectric constant (a tensor quantity due to the anisotropy of the 2D material), φ is the electric potential, n_D is the density of dopants within the semiconductor, ϵ_0 is the vacuum dielectric constant, and $k_b T$ is Boltzmann's constant times temperature. $\boldsymbol{D}$ is the diffusion coefficient (again a tensor), t_c is the carrier lifetime, α is the absorption coefficient of the semiconductor, P_0 is the power in the gaussian excitation beam, σ_0 is the beam standard deviation, x_g/y_g denote the excitation centroid, and z denotes the vertical position within the sheet.

These equations were implemented within COMSOL using general PDE interfaces. Simulations of basal plane experiments employed a geometry consisting of an oblate cylinder with a diameter of 30 µm. Step-edge simulations employed a similar geometry with a rectangular volume removed to approximate a step in the center of the geometry. The following boundary conditions were employed to find solutions to Poisson's equation:

$$\varphi(z = 0) = 0 \qquad S38$$

$$\varphi\left(\left[x - x_p\right]^2 + \left[y - y_p\right]^2 < r_0^2, z = w\right) = E_{fb} - E \qquad S39$$

Here, w is the nanosheet thickness at the point of pipet contact, r_0 is the pipet radius, x_p/y_p denote the lateral pipet location, and $E_{fb} - E$ represents the applied potential with respect to the flatband potential. In the step-edge simulations, an additional boundary condition was employed:

$$\left.\frac{\partial \varphi}{\partial x}\right|_{x=0, w_2 > z > w_1} = \frac{Q}{\epsilon_{xy} \epsilon_0} \qquad S40$$

where Q is the surface charge density at the defect surface. For the drift-diffusion equations, boundary conditions were employed at the pipet and defect interfaces:

$$C\left(\left[x - x_p\right]^2 + \left[y - y_p\right]^2 < r_0^2, z = w\right) = 0 \qquad S41$$

$$C(x = 0, w_2 > z > w_1) = 0 \qquad S42$$

Values of Physical Constants Employed in Finite Element Simulations

Table S1. Values of physical constants employed in finite element simulations. Subscript *z*'s and *xy*'s denote out-of-plane and in-plane quantities, respectively. Values were selected to match well to experimental measurements or typical literature values.[3–6]

Quantity	Value	Quantity	Value
ϵ_z	8	α	9×10^4 cm^{-1}
ϵ_{xy}	16	n_D	1×10^{17} cm^{-3}
ϵ_0	8.854×10^{-12} F m^{-1}	q	1.6×10^{-19} C
$E - E_{fb}$	0.5 V	k_bT	0.026 eV

References

(1) Smith, L. M.; Wake, D. R.; Wolfe, J. P.; Levi, D.; Klein, M. V.; Klem, J.; Henderson, T.; Morkoç, H. Picosecond Imaging of Photoexcited Carriers in Quantum Wells: Anomalous Lateral Confinement at High Densities. *Phys. Rev. B* **1988**, *38* (8), 5788–5791.

(2) *CRC Handbook of Chemistry and Physics*, 89th ed.; Lide, D. R., Ed.; CRC Press, 2008.

(3) Frindt, R. F. The Optical Properties of Single Crystals of WSe_2 and $MoTe_2$. *J. Phys. Chem. Solids* **1963**, *24* (9), 1107–1108.

(4) Yu, X.; Sivula, K. Photogenerated Charge Harvesting and Recombination in Photocathodes of Solvent-Exfoliated WSe_2. *Chem. Mater.* **2017**, *29* (16), 6863–6875.

(5) Jakubowicz, A.; Mahalu, D.; Wolf, M.; Wold, A.; Tenne, R. WSe_2: Optical and Electrical Properties as Related to Surface Passivation of Recombination Centers. *Phys. Rev. B* **1989**, *40* (5), 2992–3000.

(6) Laturia, A.; Van de Put, M. L.; Vandenberghe, W. G. Dielectric Properties of Hexagonal Boron Nitride and Transition Metal Dichalcogenides: From Monolayer to Bulk. *npj 2D Mater. Appl.* **2018**, *2* (1), 6.

COMSOL Model Report

1 Global Definitions

Date	Dec 6, 2020 4:42:10 PM

GLOBAL SETTINGS

Name	Step Diffusion Model SI.mph
COMSOL version	COMSOL 5.3 (Build: 223)

USED PRODUCTS

COMSOL Multiphysics

1.1 PARAMETERS 1

PARAMETERS

Name	Expression	Value	Description
rp	126[nm]	1.26E−7 m	
w	30[nm]	3E−8 m	
ws	64[nm]	6.4E−8 m	
nD	1e17[1/cm^3]	1E23 $1/m^3$	
kbT	0.0257[eV]	4.1176E−21 J	
rs	30[um]	3E−5 m	
epxy	16	16	
epz	8	8	
dPhi	0.5[V]	0.5 V	
Lxy	2.8[um]	2.8E−6 m	
RL	480	480	
Lz	Lxy/RL	5.8333E−9 m	
tau	1[ns]	1E−9 s	
Dxy	Lxy^2/tau	0.00784 m^2/s	
Dz	Lz^2/tau	3.4028E−8 m^2/s	
S	0.725[um]	7.25E−7 m	
P0	600[nW]	6E−7 W	
wl	633[nm]	6.33E−7 m	
Ep	1240[nm*eV]/wl	3.1385E−19 J	
N0	P0/Ep	1.9117E12 1/s	
alpha	9e4[1/cm]	9E6 1/m	
xp	5[um]	5E−6 m	
h0	1[nm]	1E−9 m	

Name	Expression	Value	Description
beta	0.1	0.1	
Nh	ceil(1/beta*log(1 + w/h0*(exp(beta) - 1)))	15	
xg	-3.5[um]	−3.5E−6 m	
Qs	1e-6[C/cm^2]	0.01 C/m²	
Nhs	ceil(1/beta*log(1 + ws/h0*(exp(beta) - 1)))	21	
Ny	40	40	
Nyr	40	40	

2 Component 1

COMPONENT SETTINGS

Unit system	SI

2.1 DEFINITIONS

2.1.1 Variables

Variables 1

SELECTION

Geometric entity level	Entire model

Name	Expression	Unit	Description
Rg	N0*alpha*exp(-alpha*z)/(2*pi*S^2)*exp(-1*((x - xg)^2 + y^2)/(2*S^2))	$1/(m^3 \cdot s)$	

2.1.2 Coordinate Systems

Boundary System 1

Coordinate system type	Boundary system
Tag	sys1

COORDINATE NAMES

First	Second	Third
t1	t2	n

2.2 GEOMETRY 1

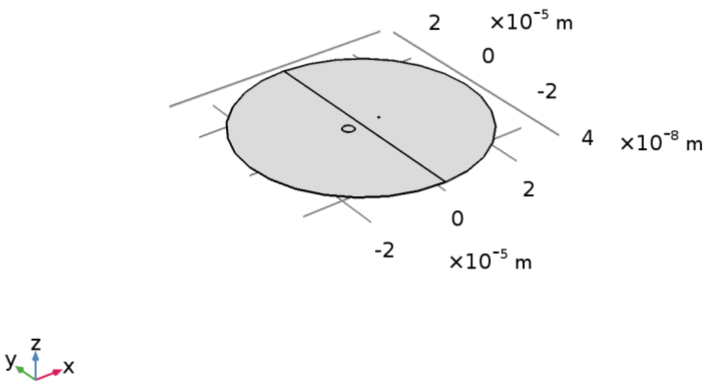

Geometry 1

UNITS

Length unit	m
Angular unit	deg

GEOMETRY STATISTICS

Description	Value
Space dimension	3
Number of domains	11
Number of boundaries	57
Number of edges	97
Number of vertices	54

2.2.1 Work Plane 1 (wp1)

UNITE OBJECTS

Description	Value
Unite objects	On

Plane Geometry (sequence2D)

Rectangle 1 (r1)

POSITION

Description	Value
Position	{-w, -rs}

SIZE

Description	Value
Width	w
Height	2*rs

Rectangle 2 (r2)

POSITION

Description	Value
Position	{0, -rs}

SIZE

Description	Value
Width	w
Height	2*rs

Circle 1 (c1)

POSITION

Description	Value
Position	{-w, 0}

ROTATION ANGLE

Description	Value
Rotation	90

SIZE AND SHAPE

Description	Value
Radius	rs
Sector angle	180

Circle 2 (c2)

POSITION

Description	Value
Position	{w, 0}

ROTATION ANGLE

Description	Value
Rotation	270

SIZE AND SHAPE

Description	Value
Radius	rs
Sector angle	180

Circle 3 (c3)

POSITION

Description	Value
Position	{xp, 0}

SIZE AND SHAPE

Description	Value
Radius	rp

Circle 4 (c4)

POSITION

Description	Value
Position	{xg, 0}

SIZE AND SHAPE

Description	Value
Radius	2*S

Circle 5 (c5)

POSITION

Description	Value
Position	{xp, 0}

SIZE AND SHAPE

Description	Value
Radius	rp + w
Sector angle	180

Circle 6 (c6)

POSITION

Description	Value
Position	{xp, 0}

ROTATION ANGLE

Description	Value
Rotation	180

SIZE AND SHAPE

Description	Value
Radius	rp + w
Sector angle	180

2.2.2 Extrude 1 (ext1)

SETTINGS

Description	Value
Work plane	Work Plane 1

DISTANCES

Distances (m)
w

SCALES

Scales xw	Scales yw
1	1

DISPLACEMENTS

Displacements xw (m)	Displacements yw (m)
0	0

TWIST ANGLES

Twist angles (deg)
0

2.2.3 Work Plane 2 (wp2)

UNITE OBJECTS

Description	Value
Unite objects	On

Plane Geometry (sequence2D)

Rectangle 1 (r1)

POSITION

Description	Value
Position	{-w, -rs}

SIZE

Description	Value
Width	w
Height	2*rs

Circle 1 (c1)

POSITION

Description	Value
Position	{-w, 0}

ROTATION ANGLE

Description	Value
Rotation	90

SIZE AND SHAPE

Description	Value
Radius	rs
Sector angle	180

Circle 4 (c4)

POSITION

Description	Value
Position	{xg, 0}

SIZE AND SHAPE

Description	Value
Radius	2*S

2.2.4 Extrude 2 (ext2)

SETTINGS

Description	Value
Work plane	Work Plane 2

DISTANCES

Distances (m)
ws

SCALES

Scales xw	Scales yw
1	1

DISPLACEMENTS

Displacements xw (m)	Displacements yw (m)
0	0

TWIST ANGLES

Twist angles (deg)
0

2.3 POISSON-BOLTZMANN

USED PRODUCTS

COMSOL Multiphysics

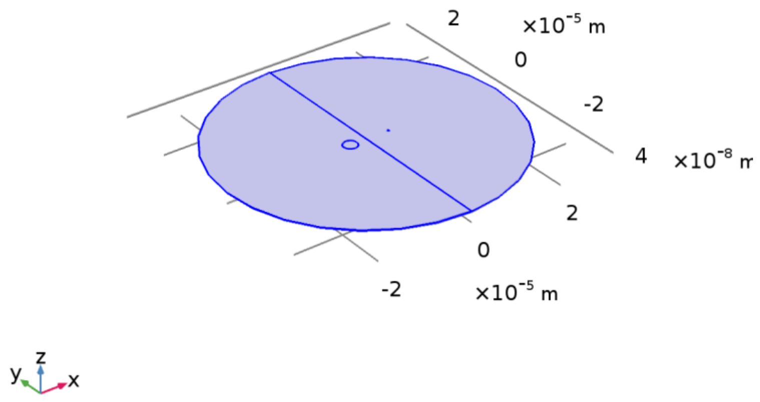

Poisson-Boltzmann

SELECTION

Geometric entity level	Domain
Selection	Domains 1–11

SETTINGS

Description	Value
Shape function type	Lagrange
Element order	Quadratic
Compute boundary fluxes	On
Apply smoothing to boundary fluxes	On
Value type when using splitting of complex variables	Complex
Frame	Spatial
Dependent variable quantity	Electric potential (V)
Source term quantity	None
Unit	C/m^3

VARIABLES

Name	Expression	Unit	Description	Selection
p.nx	nx		Normal vector, x component	Boundaries 1–57
p.ny	ny		Normal vector, y component	Boundaries 1–57
p.nz	nz		Normal vector, z component	Boundaries 1–57
p.nxmesh	root.nxmesh		Normal vector (mesh), x component	Boundaries 1–57

Name	Expression	Unit	Description	Selection
p.nymesh	root.nymesh		Normal vector (mesh), y component	Boundaries 1–57
p.nzmesh	root.nzmesh		Normal vector (mesh), z component	Boundaries 1–57

2.3.1 Coefficient Form PDE 1

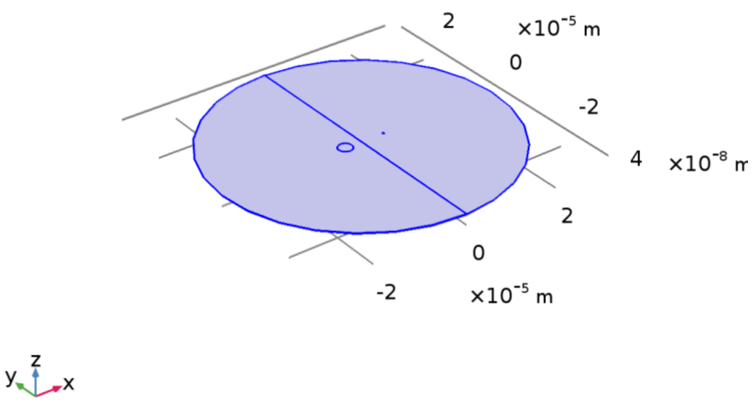

Coefficient Form PDE 1

SELECTION

Geometric entity level	Domain
Selection	Domains 1–11

EQUATIONS

$$e_a\frac{\partial^2 phi}{\partial t^2}+d_a\frac{\partial phi}{\partial t}+\nabla\cdot(-c\nabla phi-\alpha phi+\gamma)+\beta\cdot\nabla phi+a phi=f$$

$$\nabla=[\frac{\partial}{\partial x},\frac{\partial}{\partial y},\frac{\partial}{\partial z}]$$

SETTINGS

Description	Value
Diffusion coefficient	{{epxy*epsilon0_const, 0, 0}, {0, epxy*epsilon0_const, 0}, {0, 0, epz*epsilon0_const}}
Absorption coefficient	0
Source term	e_const*nD*(1 - exp(e_const*phi/kbT))
Mass coefficient	0
Damping or mass coefficient	0

Description	Value
Conservative flux convection coefficient	{0, 0, 0}
Convection coefficient	{0, 0, 0}
Conservative flux source	{0, 0, 0}

Variables

Name	Expression	Unit	Description	Selection
domflux.phix	-epxy*epsilon0_const*phix	C/m^2	Domain flux, x component	Domains 1–11
domflux.phiy	-epxy*epsilon0_const*phiy	C/m^2	Domain flux, y component	Domains 1–11
domflux.phiz	-epz*epsilon0_const*phiz	C/m^2	Domain flux, z component	Domains 1–11

Shape functions

Name	Shape function	Unit	Description	Shape frame	Selection
phi	Lagrange (Quadratic)	V	Dependent variable phi	Spatial	Domains 1–11

2.3.2 Zero Flux 1

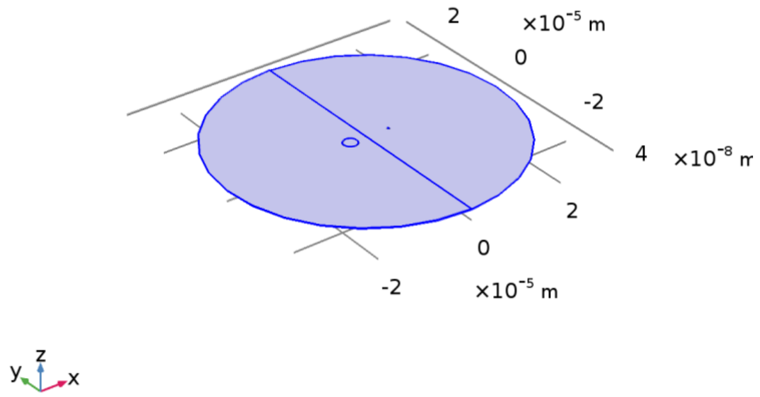

Zero Flux 1

SELECTION

Geometric entity level	Boundary
Selection	Boundaries 1–2, 4–5, 7, 10, 14, 20, 23, 25, 28–29, 31, 34–35, 37, 39, 41, 47–48

EQUATIONS

$$-\mathbf{n}\cdot(-c\nabla phi - \alpha phi + \gamma) = 0$$

$$\nabla = [\frac{\partial}{\partial x}, \frac{\partial}{\partial y}, \frac{\partial}{\partial z}]$$

2.3.3 Initial Values 1

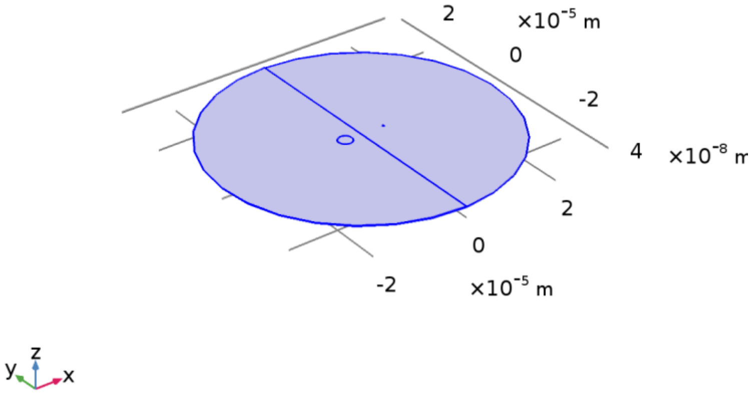

Initial Values 1

SELECTION

Geometric entity level	Domain
Selection	Domains 1–11

SETTINGS

**Description**	**Value**
Initial value for phi	0
Initial time derivative of phi	0

2.3.4 Dirichlet Boundary Condition 1

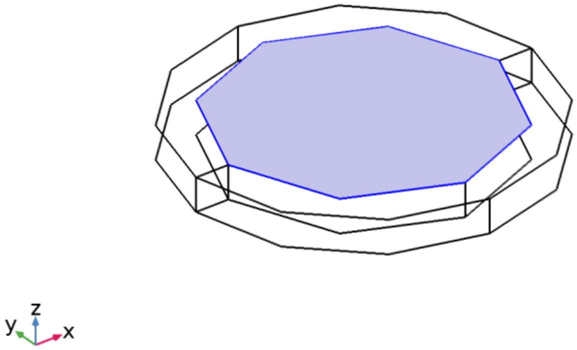

Dirichlet Boundary Condition 1

SELECTION

Geometric entity level	Boundary
Selection	Boundary 52

EQUATIONS

$$phi = r$$

$$g_{reaction} = -\mu$$

SETTINGS

Description	Value
Value on boundary	-dPhi
Prescribed value of phi	On
Apply reaction terms on	Individual dependent variables
Use weak constraints	Off
Constraint method	Elemental

Shape functions

Constraint	Constraint force	Shape function	Selection
-dPhi-phi	-test(phi)	Lagrange (Quadratic)	Boundary 52

2.3.5 Dirichlet Boundary Condition 2

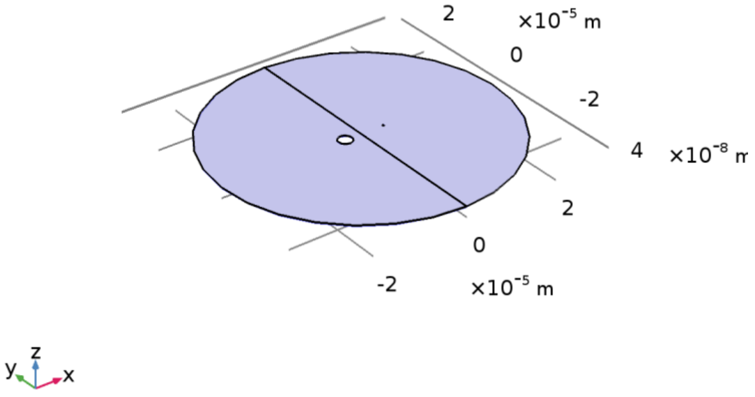

Dirichlet Boundary Condition 2

SELECTION

Geometric entity level	Boundary
Selection	Boundaries 3, 21, 32, 38, 45–46, 51

EQUATIONS

$$phi = r$$

$$g_{reaction} = -\mu$$

SETTINGS

**Description**	**Value**
Value on boundary	0
Prescribed value of phi	On
Apply reaction terms on	Individual dependent variables
Use weak constraints	Off
Constraint method	Elemental

Shape functions

**Constraint**	**Constraint force**	**Shape function**	**Selection**
-phi	-test(phi)	Lagrange (Quadratic)	Boundaries 3, 21, 32, 38, 45–46, 51

2.3.6 Flux/Source 1

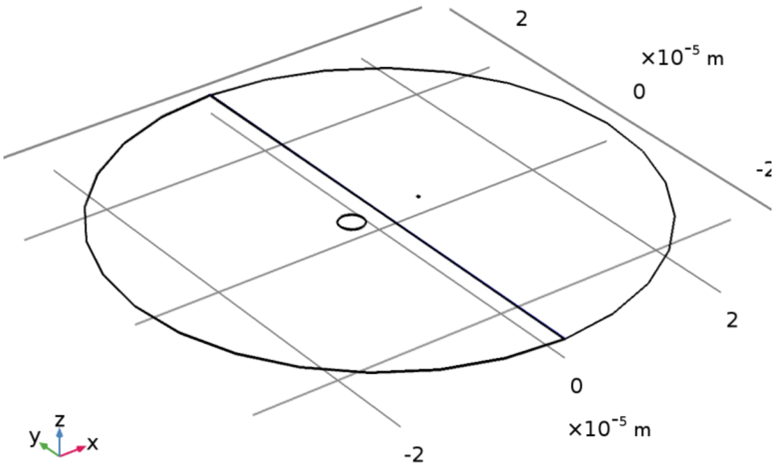

Flux/Source 1

SELECTION

Geometric entity level	Boundary
Selection	Boundary 33

EQUATIONS

$$-\mathbf{n}\cdot(-c\nabla phi - \alpha phi + \gamma) = g - qphi$$

$$\nabla = [\frac{\partial}{\partial x},\frac{\partial}{\partial y},\frac{\partial}{\partial z}]$$

SETTINGS

**Description**	**Value**
Boundary flux/source	Qs
Boundary absorption/impedance term	0

Variables

**Name**	**Expression**	**Unit**	**Description**	**Selection**
p.g_phi	Qs	C/m^2	Boundary flux/source	Boundary 33

2.4 DRIFT-DIFFUSION

USED PRODUCTS

COMSOL Multiphysics

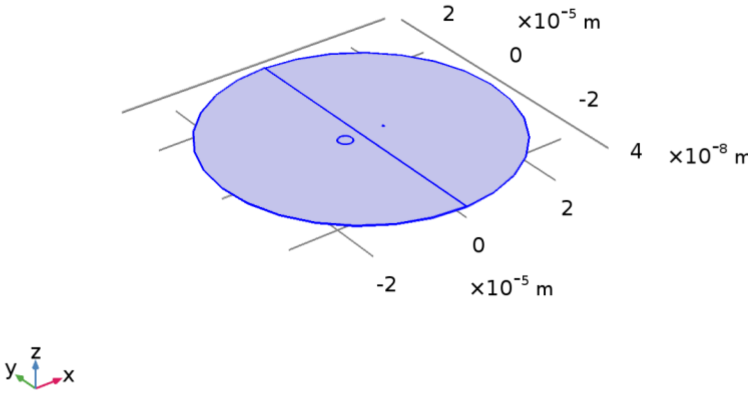

Drift-Diffusion

SELECTION

Geometric entity level	Domain
Selection	Domains 1–11

SETTINGS

**Description**	**Value**
Shape function type	Lagrange
Element order	Quadratic
Compute boundary fluxes	On
Apply smoothing to boundary fluxes	On
Value type when using splitting of complex variables	Complex
Frame	Spatial
Dependent variable quantity	Number density (1/m^3)
Source term quantity	None
Unit	m^ - 3*s^ - 1

VARIABLES

**Name**	**Expression**	**Unit**	**Description**	**Selection**
C.nx	nx		Normal vector, x component	Boundaries 1–57
C.ny	ny		Normal vector, y component	Boundaries 1–57
C.nz	nz		Normal vector, z component	Boundaries 1–57
C.nxmesh	root.nxmesh		Normal vector (mesh), x component	Boundaries 1–57

Name	Expression	Unit	Description	Selection
C.nymesh	root.nymesh		Normal vector (mesh), y component	Boundaries 1–57
C.nzmesh	root.nzmesh		Normal vector (mesh), z component	Boundaries 1–57

2.4.1 Coefficient Form PDE 1

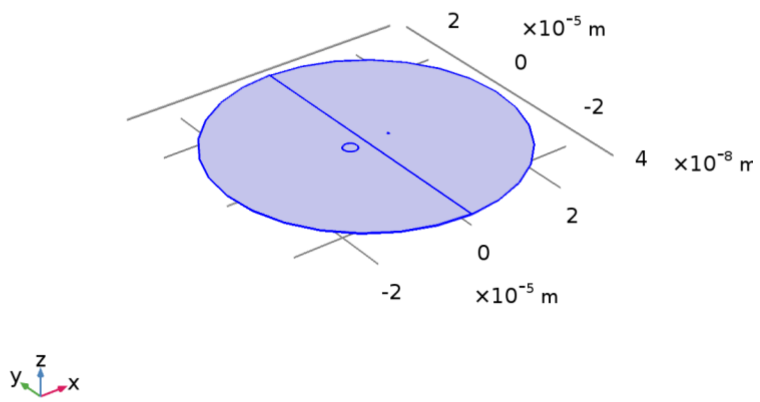

Coefficient Form PDE 1

SELECTION

Geometric entity level	Domain
Selection	Domains 1–11

EQUATIONS

$$e_a\frac{\partial^2 Ch}{\partial t^2}+d_a\frac{\partial Ch}{\partial t}+\nabla\cdot(-c\nabla Ch-\alpha Ch+\gamma)+\beta\cdot\nabla Ch+aCh=f$$

$$\nabla=[\frac{\partial}{\partial x},\frac{\partial}{\partial y},\frac{\partial}{\partial z}]$$

SETTINGS

Description	Value
Diffusion coefficient	{{Dxy, 0, 0}, {0, Dxy, 0}, {0, 0, Dz}}
Absorption coefficient	1/tau
Source term	Rg
Mass coefficient	0
Damping or mass coefficient	1

Description	Value
Conservative flux convection coefficient	{e_const*Dxy*phix/kbT, e_const*Dxy*phiy/kbT, e_const*Dz*phiz/kbT}
Convection coefficient	{0, 0, 0}
Conservative flux source	{0, 0, 0}

Variables

Name	Expression	Unit	Description	Selection
domflux.Chx	Dxy*(-Chx-e_const*phix*Ch/kbT)	$1/(m^2\cdot s)$	Domain flux, x component	Domains 1–11
domflux.Chy	Dxy*(-Chy-e_const*phiy*Ch/kbT)	$1/(m^2\cdot s)$	Domain flux, y component	Domains 1–11
domflux.Chz	Dz*(-Chz-e_const*phiz*Ch/kbT)	$1/(m^2\cdot s)$	Domain flux, z component	Domains 1–11

Shape functions

Name	Shape function	Unit	Description	Shape frame	Selection
Ch	Lagrange (Quadratic)	$1/m^3$	Dependent variable Ch	Spatial	Domains 1–11

2.4.2 Zero Flux 1

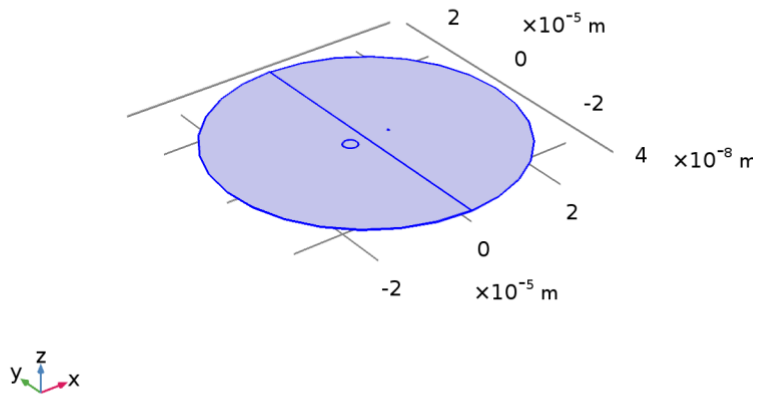

Zero Flux 1

SELECTION

Geometric entity level	Boundary
Selection	Boundaries 1–5, 7, 10, 14, 20–21, 23, 25, 28–29, 31–32, 34–35, 37–39, 41, 45–48, 51

EQUATIONS

$$-\mathbf{n}\cdot(-c\nabla Ch-\alpha Ch+\gamma)=0$$

$$\nabla=[\frac{\partial}{\partial x},\frac{\partial}{\partial y},\frac{\partial}{\partial z}]$$

2.4.3 Initial Values 1

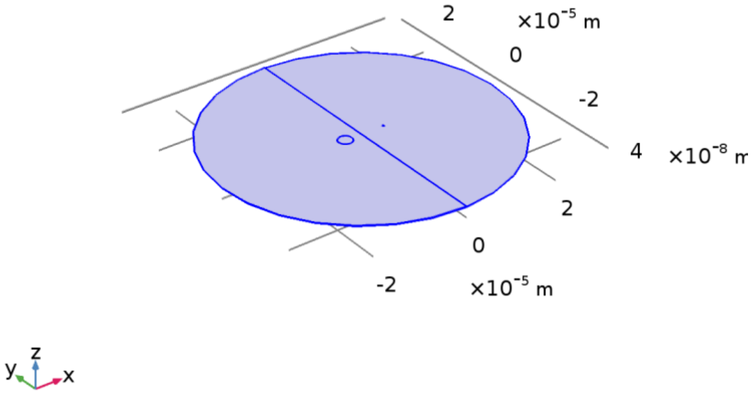

Initial Values 1

SELECTION

Geometric entity level	Domain
Selection	Domains 1–11

SETTINGS

Description	Value
Initial value for Ch	Rg*tau
Initial time derivative of Ch	0

2.4.4 Dirichlet Boundary Condition 1

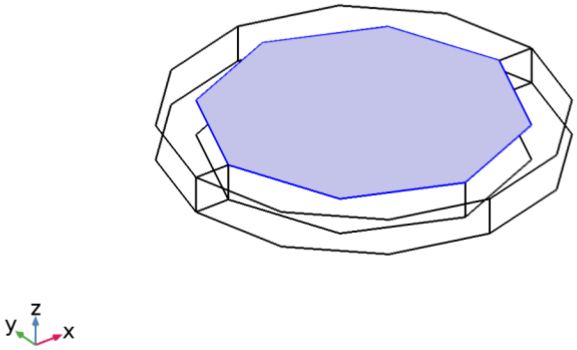

Dirichlet Boundary Condition 1

SELECTION

Geometric entity level	Boundary
Selection	Boundary 52

EQUATIONS

$$Ch = r$$
$$g_{reaction} = -\mu$$

SETTINGS

Description	Value
Value on boundary	0
Prescribed value of Ch	On
Apply reaction terms on	Individual dependent variables
Use weak constraints	Off
Constraint method	Elemental

Shape functions

Constraint	Constraint force	Shape function	Selection
-Ch	-test(Ch)	Lagrange (Quadratic)	Boundary 52

2.4.5 Dirichlet Boundary Condition 2

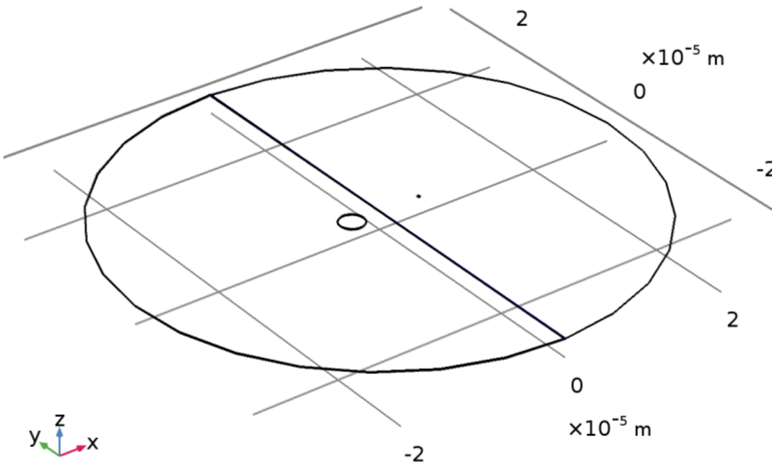

Dirichlet Boundary Condition 2

SELECTION

Geometric entity level	Boundary
Selection	Boundary 33

EQUATIONS

$$Ch = r$$
$$g_{reaction} = -\mu$$

SETTINGS

Description	Value
Value on boundary	0
Prescribed value of Ch	On
Apply reaction terms on	Individual dependent variables
Use weak constraints	Off
Constraint method	Elemental

Shape functions

Constraint	Constraint force	Shape function	Selection
-Ch	-test(Ch)	Lagrange (Quadratic)	Boundary 33

2.5 MESH 1

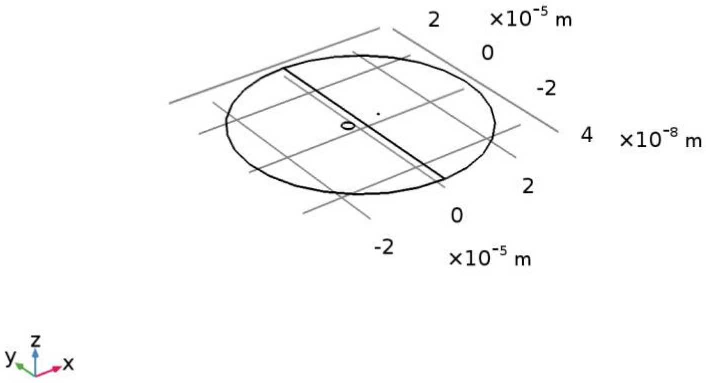

Mesh 1

2.5.1 Size (size)

SETTINGS

Description	Value
Maximum element size	6.01E-6
Minimum element size	1.08E-6
Curvature factor	0.6
Resolution of narrow regions	0.5
Maximum element growth rate	1.5

2.5.2 Size 1 (size1)

SELECTION

Geometric entity level	Domain
Selection	Geometry geom1

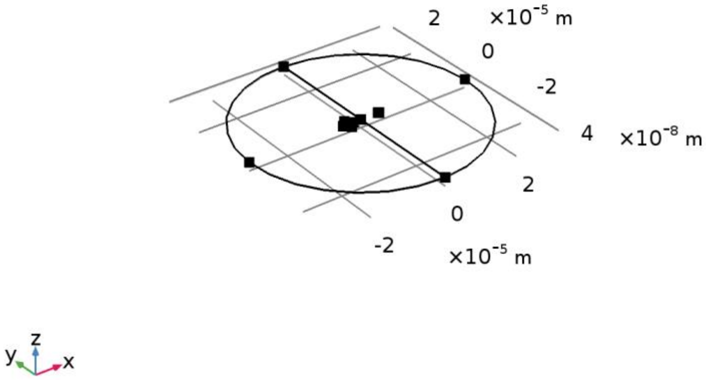

Size 1

SETTINGS

Description	Value
Maximum element size	6.01E-6
Minimum element size	1.08E-6
Curvature factor	0.6
Resolution of narrow regions	0.5
Maximum element growth rate	1.5

2.5.3 Mapped 1 (map1)

SELECTION

Geometric entity level	Boundary
Selection	Boundaries 47–48

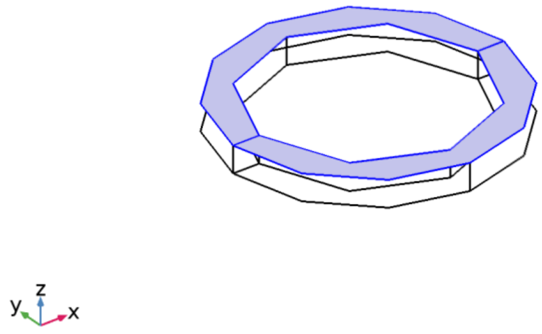

Mapped 1

Distribution 1 (dis1)

SELECTION

Geometric entity level	Edge
Selection	Edges 79–80, 86, 89

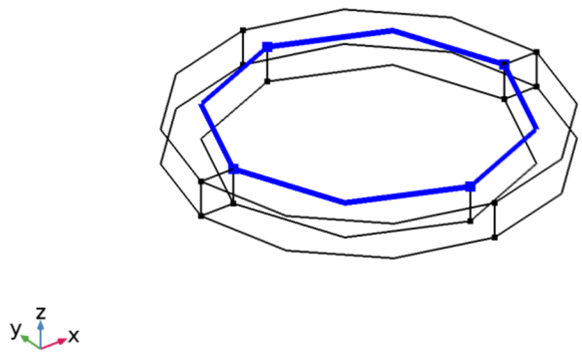

Distribution 1

SETTINGS

**Description**	**Value**
Number of elements	3

Distribution 2 (dis2)

SELECTION

Geometric entity level	Edge
Selection	Edge 73

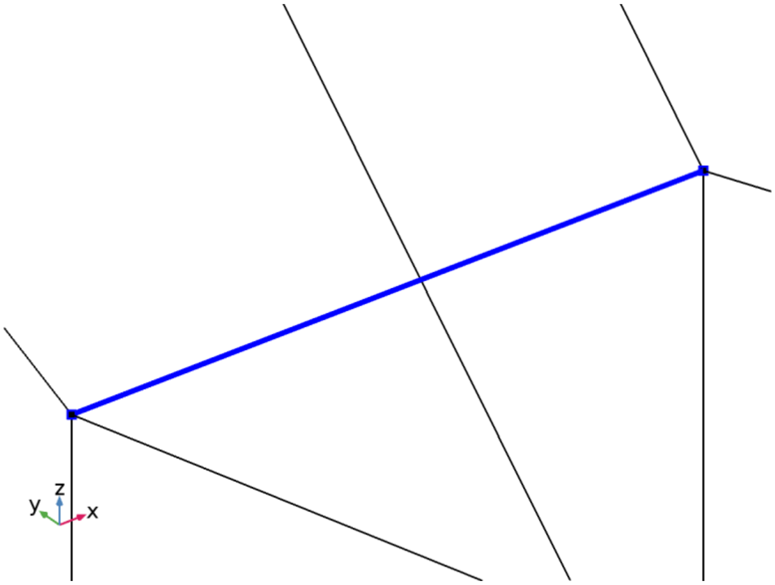

Distribution 2

SETTINGS

**Description**	**Value**
Distribution properties	Explicit distribution
Explicit element distribution	{0, 0.03333333333333333, 0.07017236393585487, 0.11088578920786052, 0.15588108279372723, 0.20560857271510286, 0.2605659484051071, 0.3213032417514574, 0.3884283320004732, 0.4626130296168887, 0.5445997999887868, 0.6352091942707551, 0.7353480617356363, 0.8460186258268543, 0.9683285147474958, 1}

Distribution 3 (dis3)

SELECTION

Geometric entity level	Edge
Selection	Edge 95

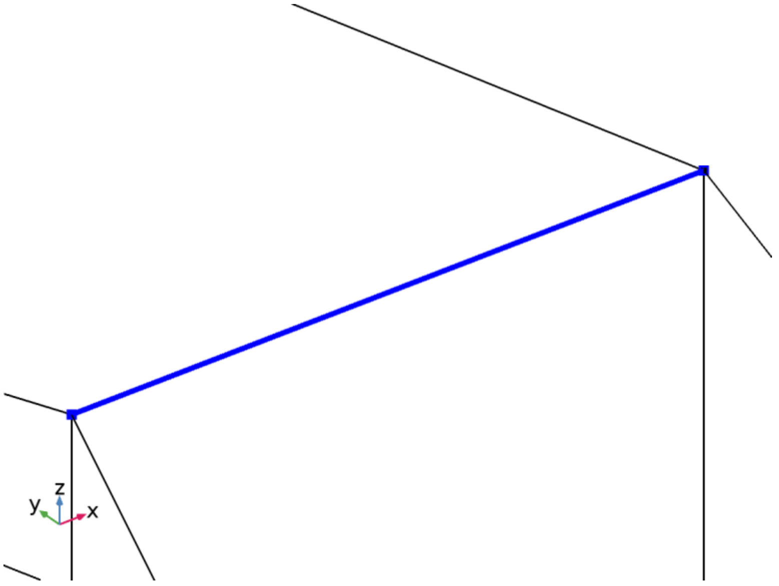

Distribution 3

SETTINGS

Description	Value
Distribution properties	Explicit distribution
Explicit element distribution	{0, 0.03333333333333333, 0.07017236393585487, 0.11088578920786052, 0.15588108279372723, 0.20560857271510286, 0.2605659484051071, 0.3213032417514574, 0.3884283320004732, 0.4626130296168887, 0.5445997999887868, 0.6352091942707551, 0.7353480617356363, 0.8460186258268543, 0.9683285147474958, 1}

2.5.4 Mapped 2 (map2)

SELECTION

Geometric entity level	Boundary
Selection	Boundary 34

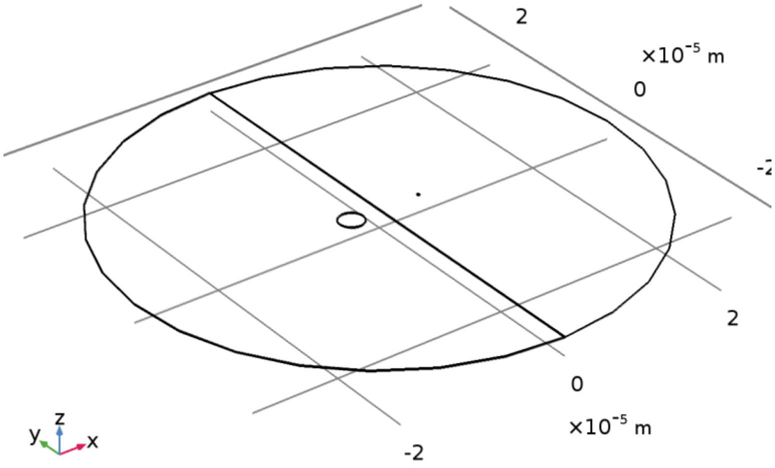

Mapped 2

Distribution 2 (dis2)

SELECTION

Geometric entity level	Edge
Selection	Edge 52

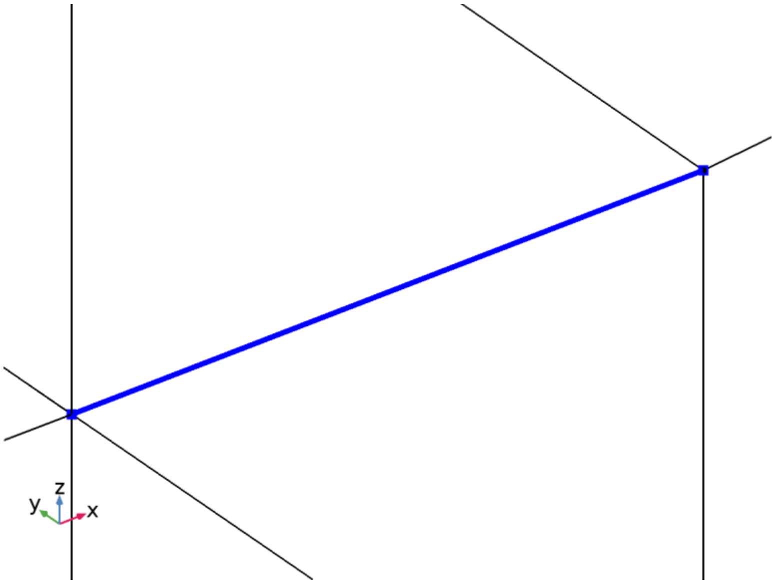

Distribution 2

SETTINGS

**Description**	**Value**
Distribution properties	Explicit distribution

Description	Value
Explicit element distribution	{0, 0.03333333333333333, 0.07017236393585487, 0.11088578920786052, 0.15588108279372723, 0.20560857271510286, 0.2605659484051071, 0.3213032417514574, 0.3884283320004732, 0.4626130296168887, 0.5445997999887868, 0.6352091942707551, 0.7353480617356363, 0.8460186258268543, 0.9683285147474958, 1}
Reverse direction	On

Distribution 3 (dis3)

SELECTION

Geometric entity level	Edge
Selection	Edge 61

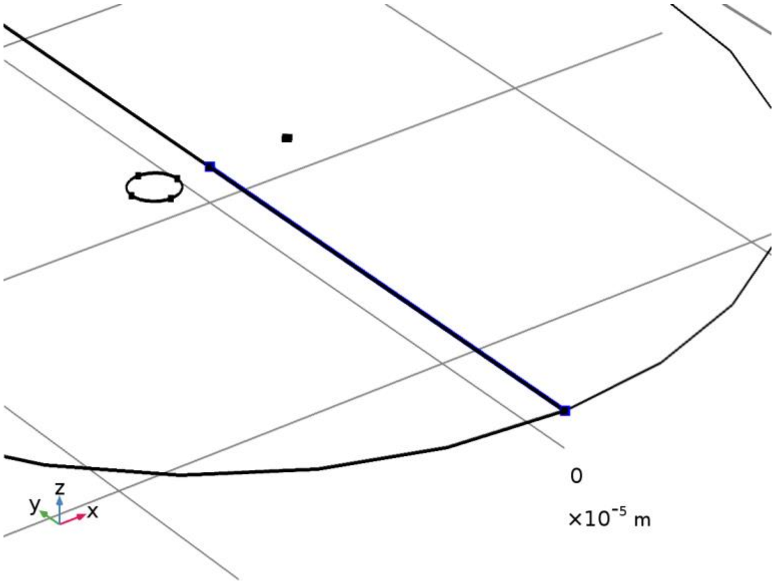

Distribution 3

SETTINGS

Description	Value
Distribution properties	Predefined distribution type
Number of elements	Ny
Element ratio	Nyr
Distribution method	Geometric sequence

Distribution 4 (dis4)

SELECTION

Geometric entity level	Edge
Selection	Edge 65

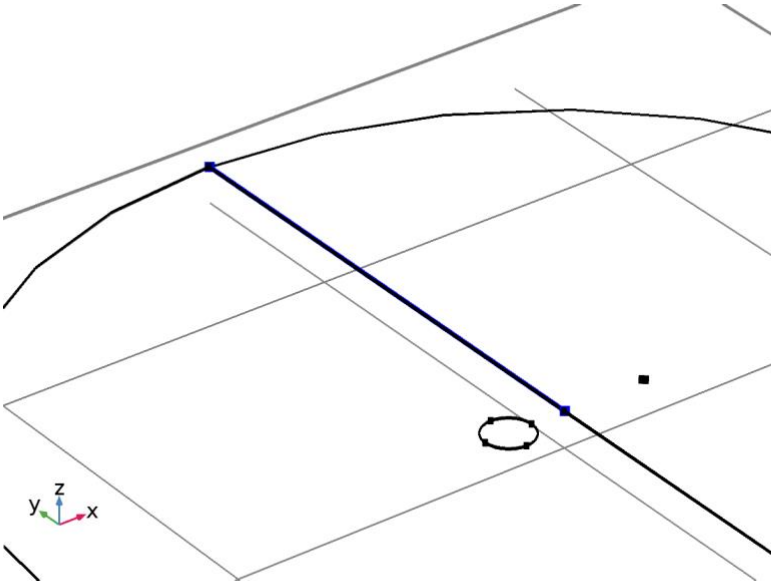

Distribution 4

SETTINGS

**Description**	**Value**
Distribution properties	Predefined distribution type
Number of elements	Ny
Element ratio	Nyr
Distribution method	Geometric sequence

Distribution 5 (dis5)

SELECTION

Geometric entity level	Edge
Selection	Edge 51

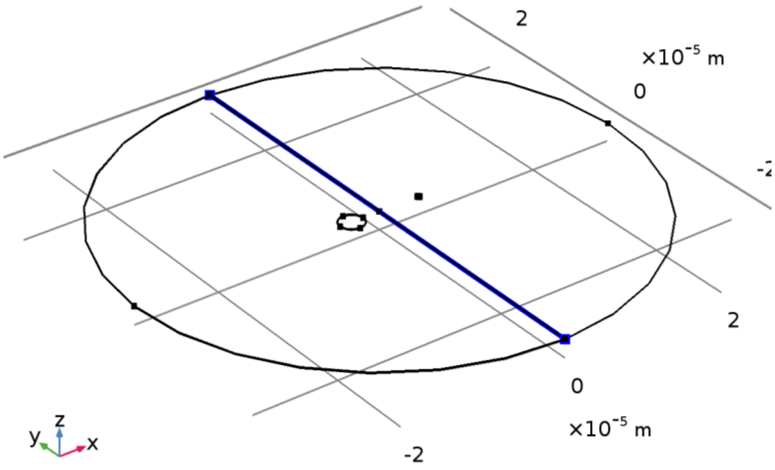

Distribution 5

SETTINGS

Description	Value
Distribution properties	Predefined distribution type
Number of elements	2*Ny
Element ratio	Nyr
Distribution method	Geometric sequence
Symmetric distribution	On
Reverse direction	On

2.5.5 Free Triangular 1 (ftri1)

SELECTION

Geometric entity level	Boundary
Selection	Boundaries 39, 52

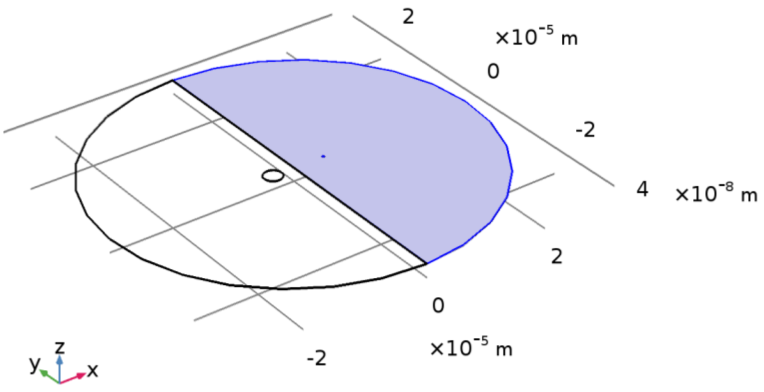

Free Triangular 1

Distribution 1 (dis1)

SELECTION

Geometric entity level	Edge
Selection	Edges 62, 68

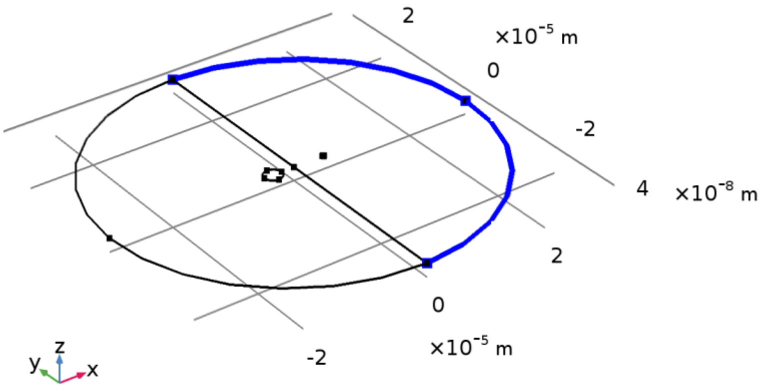

Distribution 1

SETTINGS

**Description**	**Value**
Number of elements	3

Size 1 (size1)

SELECTION

Geometric entity level	Boundary
Selection	Boundaries 39, 52

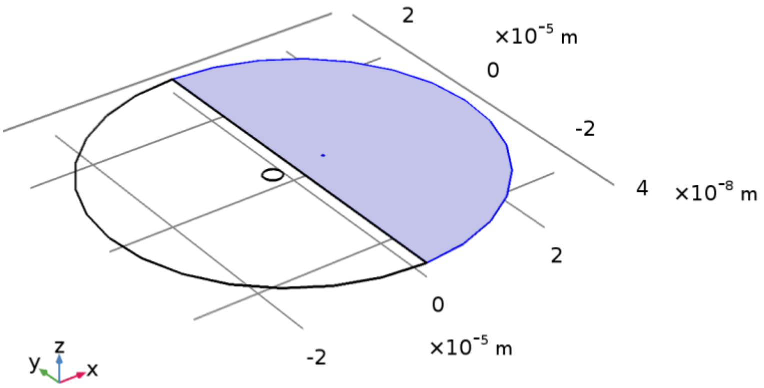

Size 1

SETTINGS

Description	Value
Maximum element size	6.01E-6
Maximum element size	Off
Minimum element size	1.08E-6
Minimum element size	Off
Curvature factor	0.6
Curvature factor	Off
Resolution of narrow regions	0.5
Resolution of narrow regions	Off
Maximum element growth rate	1.25
Custom element size	Custom

2.5.6 Swept 3 (swe3)

SELECTION

Geometric entity level	Domain
Selection	Domains 7–11

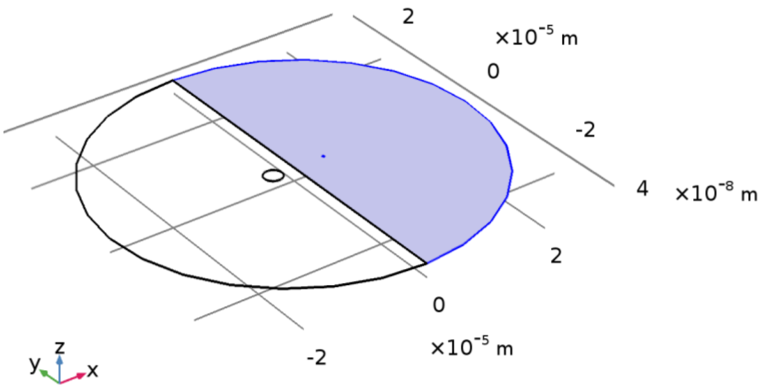

Swept 3

Distribution 1 (dis1)

SELECTION

Geometric entity level	Domain
Selection	Domains 7–11

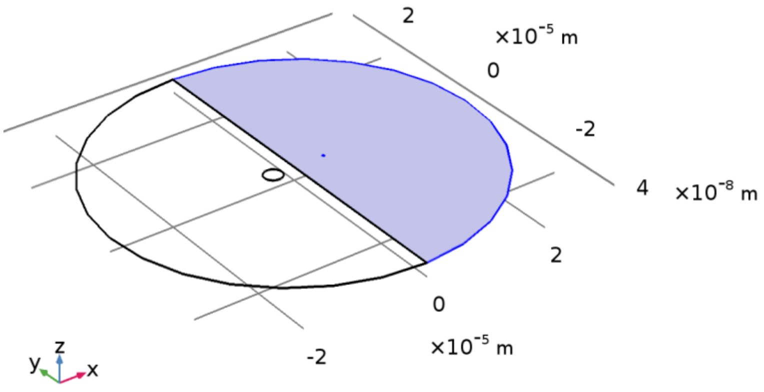

Distribution 1

SETTINGS

**Description**	**Value**
Distribution properties	Explicit distribution

Description	Value
Explicit element distribution	{0, 0.03333333333333333, 0.07017236393585487, 0.11088578920786052, 0.15588108279372723, 0.20560857271510286, 0.2605659484051071, 0.3213032417514574, 0.3884283320004732, 0.4626130296168887, 0.5445997999887868, 0.6352091942707551, 0.7353480617356363, 0.8460186258268543, 0.9683285147474958, 1}

2.5.7 Mapped 3 (map3)

SELECTION

Geometric entity level	Boundary
Selection	Boundary 24

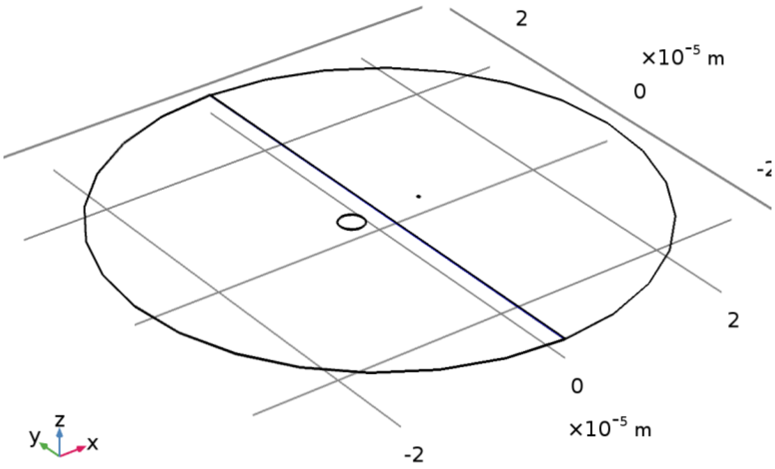

Mapped 3

Distribution 1 (dis1)

SELECTION

Geometric entity level	Edge
Selection	Edge 34

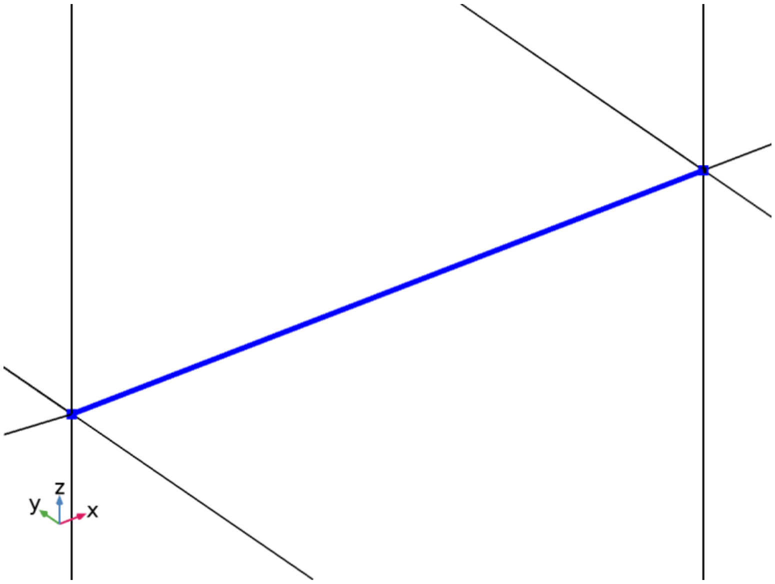

Distribution 1

SETTINGS

Description	Value
Distribution properties	Explicit distribution
Explicit element distribution	{0, 0.03333333333333333, 0.07017236393585487, 0.11088578920786052, 0.15588108279372723, 0.20560857271510286, 0.2605659484051071, 0.3213032417514574, 0.3884283320004732, 0.4626130296168887, 0.5445997999887868, 0.6352091942707551, 0.7353480617356363, 0.8460186258268543, 0.9683285147474958, 1}

Distribution 2 (dis2)

SELECTION

Geometric entity level	Edge
Selection	Edge 33

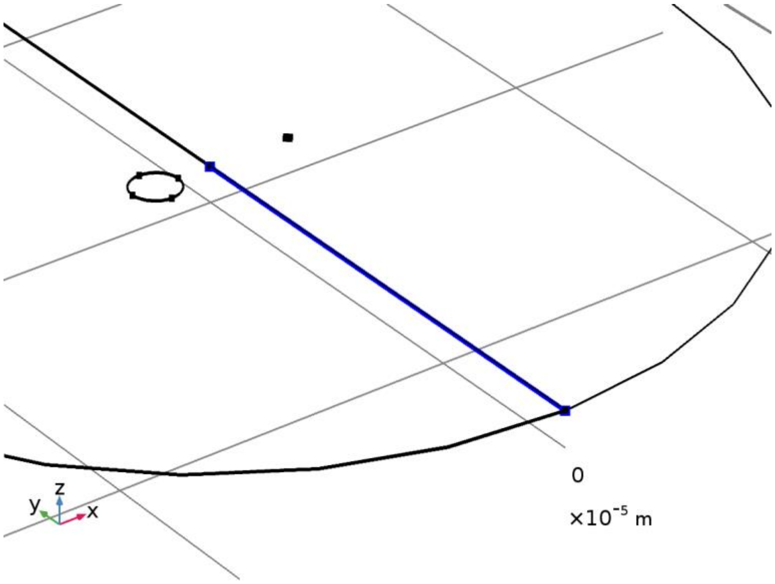

Distribution 2

SETTINGS

Description	Value
Distribution properties	Predefined distribution type
Number of elements	Ny
Element ratio	Nyr
Distribution method	Geometric sequence

Distribution 3 (dis3)

SELECTION

Geometric entity level	Edge
Selection	Edge 40

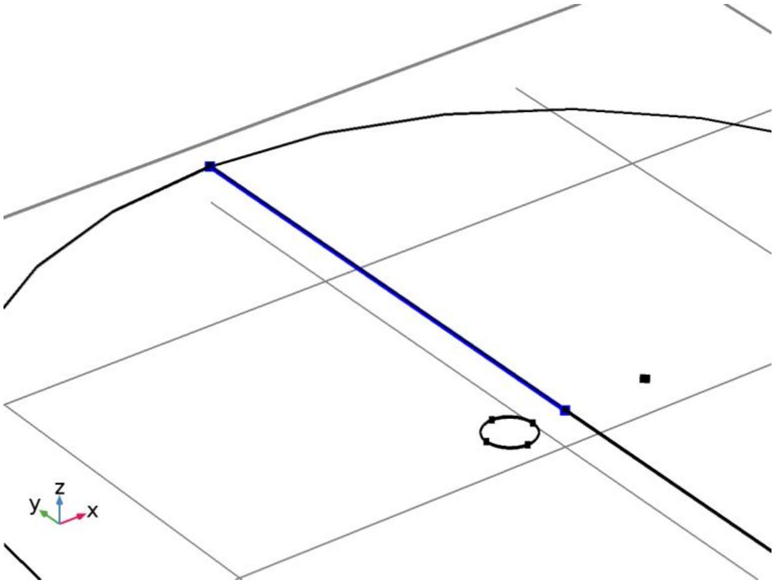

Distribution 3

SETTINGS

Description	Value
Distribution properties	Predefined distribution type
Number of elements	Ny
Element ratio	Nyr
Distribution method	Geometric sequence

2.5.8 Free Triangular 2 (ftri2)

SELECTION

Geometric entity level	Boundary
Selection	Boundaries 6, 13

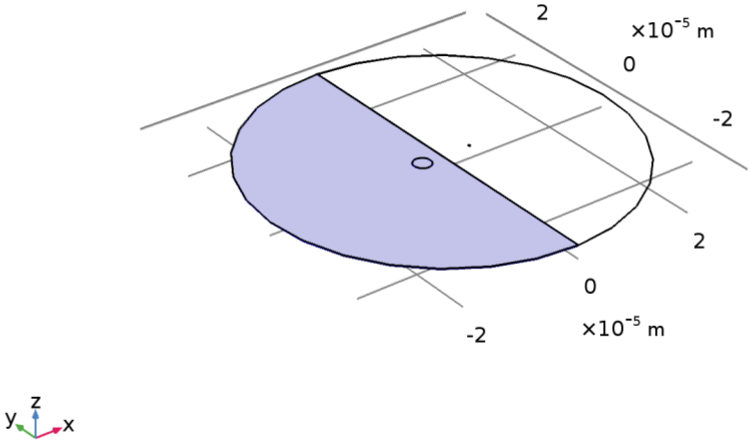

Free Triangular 2

Distribution 1 (dis1)

SELECTION

Geometric entity level	Edge
Selection	Edges 5–6, 13–14, 20, 25

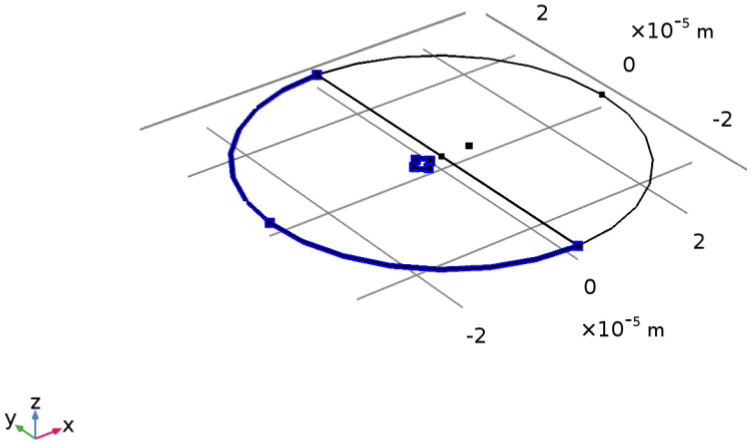

Distribution 1

SETTINGS

**Description**	**Value**
Number of elements	3

Size 1 (size1)

SELECTION

Geometric entity level	Boundary
Selection	No boundaries

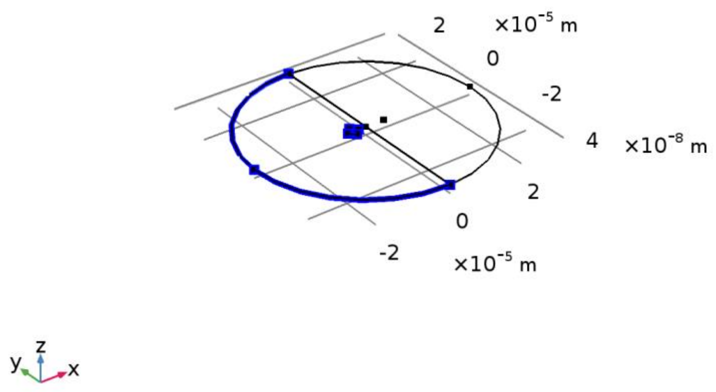

Size 1

SETTINGS

**Description**	**Value**
Maximum element size	6.0E-6
Maximum element size	Off
Minimum element size	1.08E-6
Minimum element size	Off
Curvature factor	0.6
Curvature factor	Off
Resolution of narrow regions	0.5
Resolution of narrow regions	Off
Maximum element growth rate	1.25
Custom element size	Custom

2.5.9 Swept 1 (swe1)

SELECTION

Geometric entity level	Domain
Selection	Domains 1, 3, 5

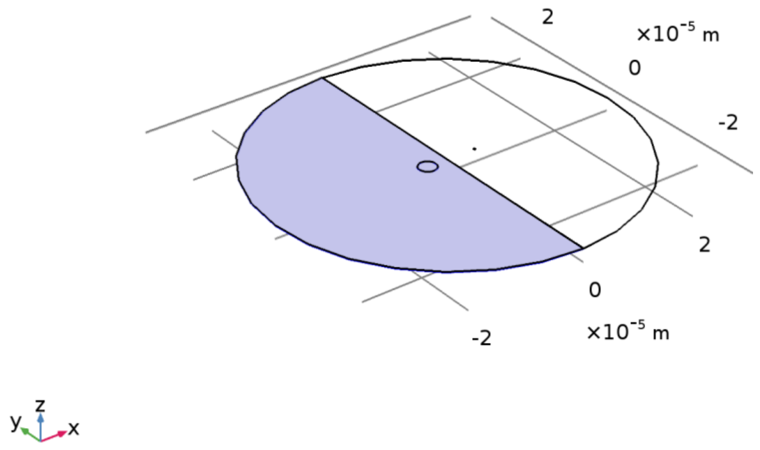

Swept 1

Distribution 1 (dis1)

SELECTION

Geometric entity level	Domain
Selection	Domains 1, 5

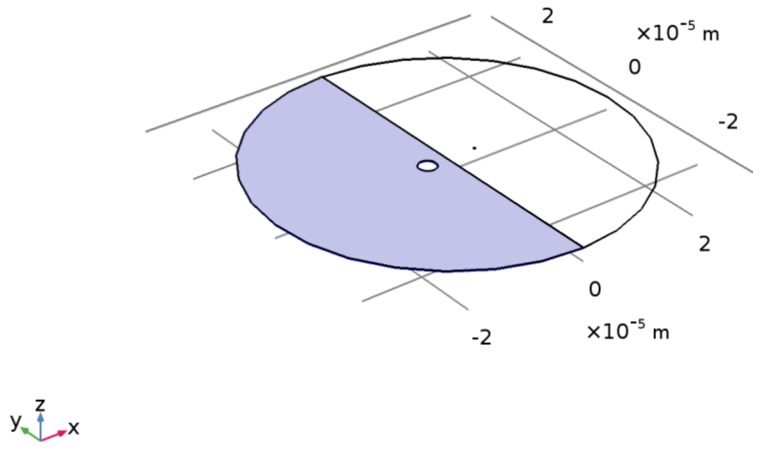

Distribution 1

SETTINGS

Description	Value
Distribution properties	Explicit distribution

Description	Value
Explicit element distribution	{0, 0.03333333333333333, 0.07017236393585487, 0.11088578920786052, 0.15588108279372723, 0.20560857271510286, 0.2605659484051071, 0.3213032417514574, 0.3884283320004732, 0.4626130296168887, 0.5445997999887868, 0.6352091942707551, 0.7353480617356363, 0.8460186258268543, 0.9683285147474958, 1}

2.5.10 Swept 2 (swe2)

SELECTION

Geometric entity level	Domain
Selection	Remaining

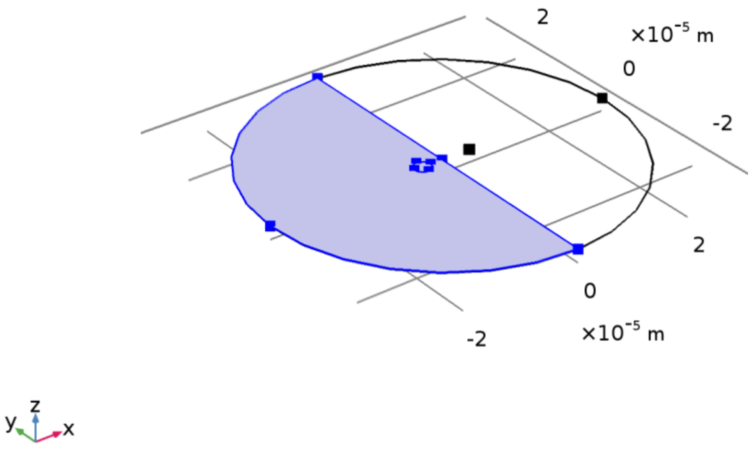

Swept 2

Distribution 1 (dis1)

SELECTION

Geometric entity level	Domain
Selection	Domain 6

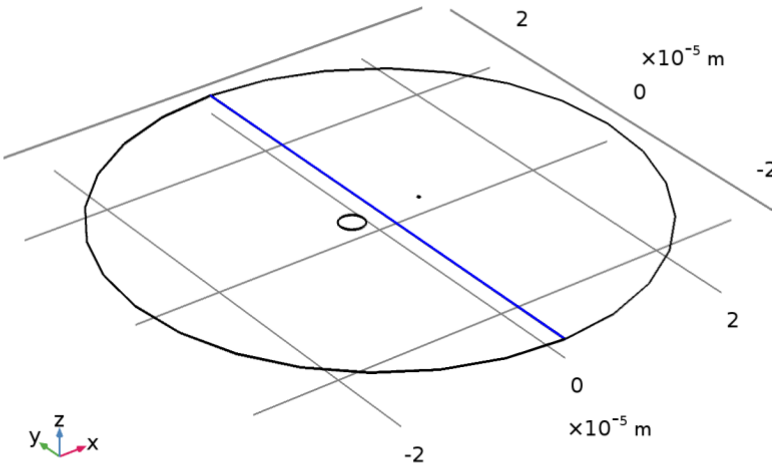

Distribution 1

SETTINGS

Description	Value
Distribution properties	Explicit distribution
Explicit element distribution	{0, 0.015625, 0.03289329559493197, 0.05197771369118462, 0.07306925755955963, 0.09637901846020447, 0.12214028831489397, 0.15061089457099563, 0.18207578062522184, 0.2168498576329166, 0.25528115624474385, 0.2977543098144165, 0.3446944039385795, 0.39657123085633794, 0.45390399128788866, 0.5172664907698366, 0.5872928824938688, 0.6646840141250425, 0.7502144421207798, 0.8447401837522321, 0.9492072844128455, 1}

3 Study 1

COMPUTATION INFORMATION

Computation time	27 h 17 min 6 s
CPU	Intel(R) Xeon(R) CPU E3-1270 v5 @ 3.60GHz, 4 cores
Operating system	Windows 7

3.1 PIPET TRANSLATION

Parameter name	Parameter value list	Parameter unit
xp	range(1,1,15)	um

STUDY SETTINGS

Description	Value
Sweep type	Specified combinations
Parameter name	xp
Parameter value list	range(1, 1, 15)
Unit	um

3.2 STATIONARY

STUDY SETTINGS

Description	Value
Include geometric nonlinearity	Off

PHYSICS AND VARIABLES SELECTION

Physics interface	Discretization
Poisson-Boltzmann (p)	physics
Drift-Diffusion (C)	physics

MESH SELECTION

Geometry	Mesh
Geometry 1 (geom1)	mesh1

STUDY EXTENSIONS

Description	Value
Auxiliary sweep	On
Sweep type	Specified combinations
Parameter value list	range(0, -1, -8)

3.3 SOLVER CONFIGURATIONS

3.3.1 Solution 1

Compile Equations: Stationary (st1)

STUDY AND STEP

Description	Value
Use study	Study 1
Use study step	Stationary

Dependent Variables 1 (v1)

GENERAL

Description	Value
Defined by study step	Stationary

INITIAL VALUE CALCULATION CONSTANTS

Description	Value
Parameter initial value list	range(0, -1, -8)[mC/m^2]

Dependent variable Ch (comp1.Ch) (comp1_Ch)

GENERAL

Description	Value
Field components	comp1.Ch

Dependent variable phi (comp1.phi) (comp1_phi)

GENERAL

Description	Value
Field components	comp1.phi

Stationary Solver 1 (s1)

GENERAL

Description	Value
Defined by study step	Stationary

RESULTS WHILE SOLVING

Description	Value
Probes	None

Parametric 1 (p1)

GENERAL

Description	Value
Defined by study step	Stationary
Parameter value list	range(0, -1, -8)

Segregated 1 (se1)

Segregated Step 1 (ss1)

GENERAL

Description	Value
Variables	Dependent variable Ch (comp1.Ch)
Linear solver	Direct

Segregated Step 2 (ss2)

GENERAL

Description	Value
Variables	Dependent variable phi (comp1.phi)
Linear solver	Direct

3.3.2 Parametric Solutions 1

xp=1 (su1)

GENERAL

Description	Value
Solution	xp=1

xp=2 (su2)

GENERAL

Description	Value
Solution	xp=2

xp=3 (su3)

GENERAL

Description	Value
Solution	xp=3

xp=4 (su4)

GENERAL

Description	Value
Solution	xp=4

xp=5 (su5)

GENERAL

Description	Value
Solution	xp=5

xp=6 (su6)

GENERAL

Description	Value
Solution	xp=6

xp=7 (su7)

GENERAL

Description	Value
Solution	xp=7

xp=8 (su8)

GENERAL

Description	Value
Solution	xp=8

xp=9 (su9)

GENERAL

Description	Value
Solution	xp=9

xp=10 (su10)

GENERAL

Description	Value
Solution	xp=10

xp=11 (su11)

GENERAL

Description	Value
Solution	xp=11

xp=12 (su12)

GENERAL

Description	Value
Solution	xp=12

xp=13 (su13)

GENERAL

Description	Value
Solution	xp=13

xp=14 (su14)

GENERAL

Description	Value
Solution	xp=14

xp=15 (su15)

GENERAL

Description	Value
Solution	xp=15
